# A high-resolution view of the immune and stromal cell response to *Haemophilus ducreyi* infection in human volunteers

**DOI:** 10.1128/mbio.03885-24

**Published:** 2025-01-30

**Authors:** Julie A. Brothwell, Yuhui Wei, Jia Wang, Tingbo Guo, Chi Zhang, Kate R. Fortney, Rory Duplantier, Li Chen, Teresa A. Batteiger, Mark H. Kaplan, Stanley M. Spinola, Sha Cao

**Affiliations:** 1 Department of Microbiology and Immunology, Indiana University School of Medicine, Indianapolis, Indiana, USA; 2 Department of Medical and Molecular Genetics and Center for Computational Biology and Bioinformatics, Indiana University School of Medicine, Indianapolis, Indiana, USA; 3 Department of Biomedical Engineering, Oregon Health and Science University, Portland, Oregon, USA; 4 Department of Computer Science, Indiana University, Bloomington, Indiana, USA; 5 Department of Medicine, Indiana University School of Medicine, Indianapolis, Indiana, USA; 6 Department of Biostatistics, University of Florida, Gainesville, Florida, USA; 7 Department of Pathology and Laboratory Medicine, Indiana University School of Medicine, Indianapolis, Indiana, USA; 8 Department of Biostatistics and Health Data Science, Indiana University School of Medicine, Indianapolis, Indiana, USA; Rutgers The State University of New Jersey, Piscataway, New Jersey, USA

**Keywords:** *Haemophilus ducreyi*, human infection, scRNA-seq, spatial transcriptomics

## Abstract

*Haemophilus ducreyi* causes the genital ulcer disease chancroid and cutaneous ulcers in children. To study its pathogenesis, we developed a human challenge model in which we infect the skin on the upper arm of human volunteers with *H. ducreyi* to the pustular stage of disease. The model has been used to define lesional architecture, describe the immune infiltrate into the infected sites using flow cytometry, and explore the molecular basis of the immune response using bulk RNA-seq. Here, we used single cell RNA-seq (scRNA-seq) and spatial transcriptomics to simultaneously characterize multiple cell types within infected human skin and determine the cellular origin of differentially expressed transcripts that we had previously identified by bulk RNA-seq. We obtained paired biopsies of pustules and wounded (mock infected) sites from five volunteers for scRNA-seq. We identified 13 major cell types, including T- and NK-like cells, macrophages, dendritic cells, as well as other cell types typically found in the skin. Immune cell types were enriched in pustules, and some subtypes within the major cell types were exclusive to pustules. Sufficient tissue specimens for spatial transcriptomics were available from four of the volunteers. T- and NK-like cells were highly associated with multiple antigen presentation cell types. In pustules, type I interferon stimulation was high in areas that were high in antigen presentation—especially in macrophages near the abscess—compared to wounds. Together, our data provide a high-resolution view of the cellular immune response to the infection of the skin with a human pathogen.

**IMPORTANCE:**

A high-resolution view of the immune infiltrate due to infection with an extracellular bacterial pathogen in human skin has not yet been defined. Here, we used the human skin pathogen *Haemophilus ducreyi* in a human challenge model to identify on a single cell level the types of cells that are present in volunteers who fail to spontaneously clear infection and form pustules. We identified 13 major cell types. Immune cells and immune-activated stromal cells were enriched in pustules compared to wounded (mock infected) sites. Pustules formed despite the expression of multiple pro-inflammatory cytokines, such as IL-1β and type I interferon. Interferon stimulation was most evident in macrophages, which were proximal to the abscess. The pro-inflammatory response within the pustule may be tempered by regulatory T cells and cells that express indoleamine 2,3-dioxygenase, leading to failure of the immune system to clear *H. ducreyi*.

## INTRODUCTION

*Haemophilus ducreyi* causes chancroid, a genital ulcer disease endemic in resource poor communities of Africa, which facilitates the transmission of the human immunodeficiency virus (1–4). Although long thought to be exclusively sexually transmitted, *H. ducreyi* has recently been recognized as a major cause of painful exudative cutaneous ulcers in children who live in equatorial regions of the South Pacific and Africa (1, 4–8). Cutaneous ulcers are not sexually transmitted and are likely due to traumatic wounds that occur in children whose skin is colonized with *H. ducreyi* or contact of wounds with environmental reservoirs of the organism (4, 7, 9, 10). Thus, *H. ducreyi* is an important problem for global health.

To study *H. ducreyi* pathogenesis, we developed a human infection model in which healthy adult volunteers are inoculated via puncture wounds with *H. ducreyi* strain 35000HP (HP, human passaged) on the skin overlying the deltoid (reviewed in references 11–13). Within 24 hours of inoculation, papules form at inoculated sites, and either spontaneously resolve or evolve into pustules 2 to 5 days later, mimicking the initial stages of natural chancroid (12). Due to safety considerations, volunteers are only infected until the pustules become painful, usually 6 to 8 days after inoculation. During this time, neutrophils, macrophages, dendritic cells (DCs), T cells, and NK cells are recruited to the site of infection (13–18). The architecture of experimental pustules resembles a suppurative granuloma in which macrophages admixed with regulatory T cells form a collar below a neutrophilic abscess, while memory T cells and NK cells remain below the collar (13, 14). In both natural ulcers and experimental pustules, *H. ducreyi* is found in the abscess and co-localizes with macrophages and neutrophils, which fail to ingest the organism due to the secretion of anti-phagocytic proteins by *H. ducreyi* (14, 19).

We recently determined the human and *H. ducreyi* transcriptomes and metabolomes of pustules and wounded (mock inoculated) sites of infected volunteers (20). We identified changes in fatty acid metabolism and mitigation of oxidative damage that occurred during infection, which suggests that the host response to *H. ducreyi* infection is both pro- and anti-inflammatory (20). Although this study informed how *H. ducreyi* interacts with the host, the cellular sources of the differentially expressed host transcripts were unclear. Whether the pro- and anti-inflammatory transcripts originated from a single cell type or whether numerous pro- and anti-inflammatory cell types were recruited to the site of infection was also unclear.

To better understand how *H. ducreyi* interacts with the human host, we performed single cell RNA-seq (scRNA-seq) and spatial transcriptomics of pustules and wounded sites obtained from human volunteers. To our knowledge, this is the first attempt to define the human immune response to an acute infection by an extracellular bacterial pathogen in the skin and compare the immune response of an acute infection to that of wound healing using scRNA-seq and spatial transcriptomics.

## RESULTS

### Experimental *H. ducreyi* infection of human volunteers

To determine the cellular sources and cellular location of the differentially expressed host transcripts at the pustular stage of *H. ducreyi* infection, we inoculated six volunteers (four men, two women; four whites, two Asians; mean age ± SD, 32.0 ± 8.2 years old) with no history of *H. ducreyi* infection with estimated delivered doses (EDD) ranging from 41 to 85 colony forming units (CFU) of 35000HP at three sites and at one site with a buffer control on the skin overlying the deltoid in four iterations (see Table S1 in the supplemental material). Our protocol permits up to three biopsies per volunteer of pustules and wounded sites; one volunteer (483) consented to only two biopsies. Per protocol, infected sites where disease spontaneously resolved were not biopsied.

Five of the six volunteers formed pustules and underwent biopsies 7 to 8 days post infection. Biopsies of two pustules were obtained from four volunteers and processed separately for scRNA-seq and spatial transcriptomics; biopsies of the single pustule obtained from volunteer 483 and the wounded sites were split in half with a razor blade; each half was processed for scRNA-seq or spatial transcriptomics. All five volunteers contributed samples for single cell analysis. Tissue specimens from volunteer 478 were used to optimize permeabilization conditions for spatial transcriptomics and were exhausted, leaving four pairs of specimens for spatial analysis.

### scRNA-seq identifies 13 cell types in wounds and pustules

**Fig 1:**
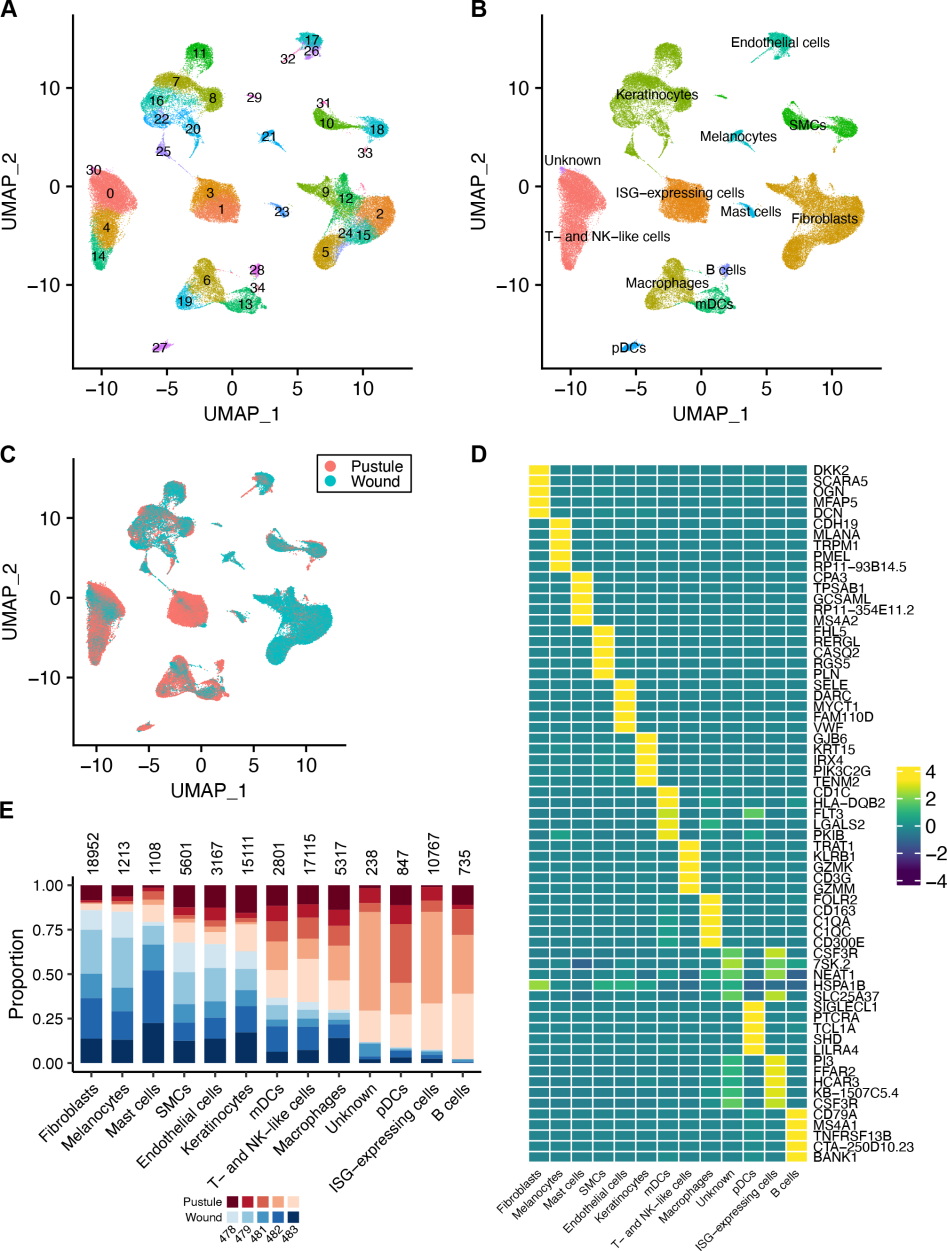
Cell types present in pustules and wounds. (**A**) Dimensional reduction UMAP analysis identified 35 cell clusters. (**B**) Clusters in panel **A** were annotated by major cell type. There were 13 major cell types. (**C**) UMAP plot showing whether an individual cell was derived from a pustule or wound. (**D**) Heatmap showing the average expression of the top 5 genes in each of the major cell types identified in panel **B**. Gene expression in rows was normalized and colored by z-score. (**E**) For each cell type in panel **B**, the proportion of cells derived from each volunteer is shown. Blue colors indicate that the cells were isolated from a wound; red colors indicate that the cells were derived from a pustule. The different shades of color indicate the volunteer number. The total number of cells belonging to each cell type is shown above each bar. SMCs, smooth muscle cells.

To explore the cellular composition of pustules and wounds, we performed scRNA-seq on pustule and wound biopsy pairs from five volunteers using the 10X Genomics Chromium platform. After quality control filtering to remove low-quality cells that had <100 or >5,000 unique features/genes, >30,000 reads, >20% mitochondrial reads, or that were predicted to be a doublet, 82,972 cells remained for analysis. Seurat (21) was used to identify variable genes, reduce dimensionality by uniform manifold approximation and projection (UMAP), and cluster cells. Thirty-five clusters were identified (Fig. 1A). Differential gene expression analysis identified genes that were unique to each cluster (Data set S1). Clusters were first annotated computationally with scType (22) and then confirmed or modified by manual annotation. We then combined clusters of the same cell type; this resulted in 13 different cell types (Fig. 1B). All 10 samples contributed to each cell cluster (Fig. 1C; Fig. S1). We identified the top 5 differentially expressed genes (DEGs) in each of the 13 cell types (Fig. 1D). The proportion of cells from pustules and wounds that contributed to each cell type was determined (Fig. 1E). There were several cell types that were primarily derived from pustules or from wounds (Fig. 1E); the proportions of fibroblasts, melanocytes, myeloid dendritic cells (mDCs), T- and NK-like (TNK) cells, macrophages, plasmacytoid dendritic cells (pDCs), and B cells were significantly different in pustule versus wound samples (all *P* < 0.05). The immune cell subsets were all higher in pustules, while the proportion of stromal cells was higher in wounds. This is consistent with the observation that, by flow cytometry, pustules contain ~10-fold the number of leukocytes than mock-infected sites (23).

### Predicted upstream regulators in bulk RNA-seq are increased in specific cell clusters

**Fig 2:**
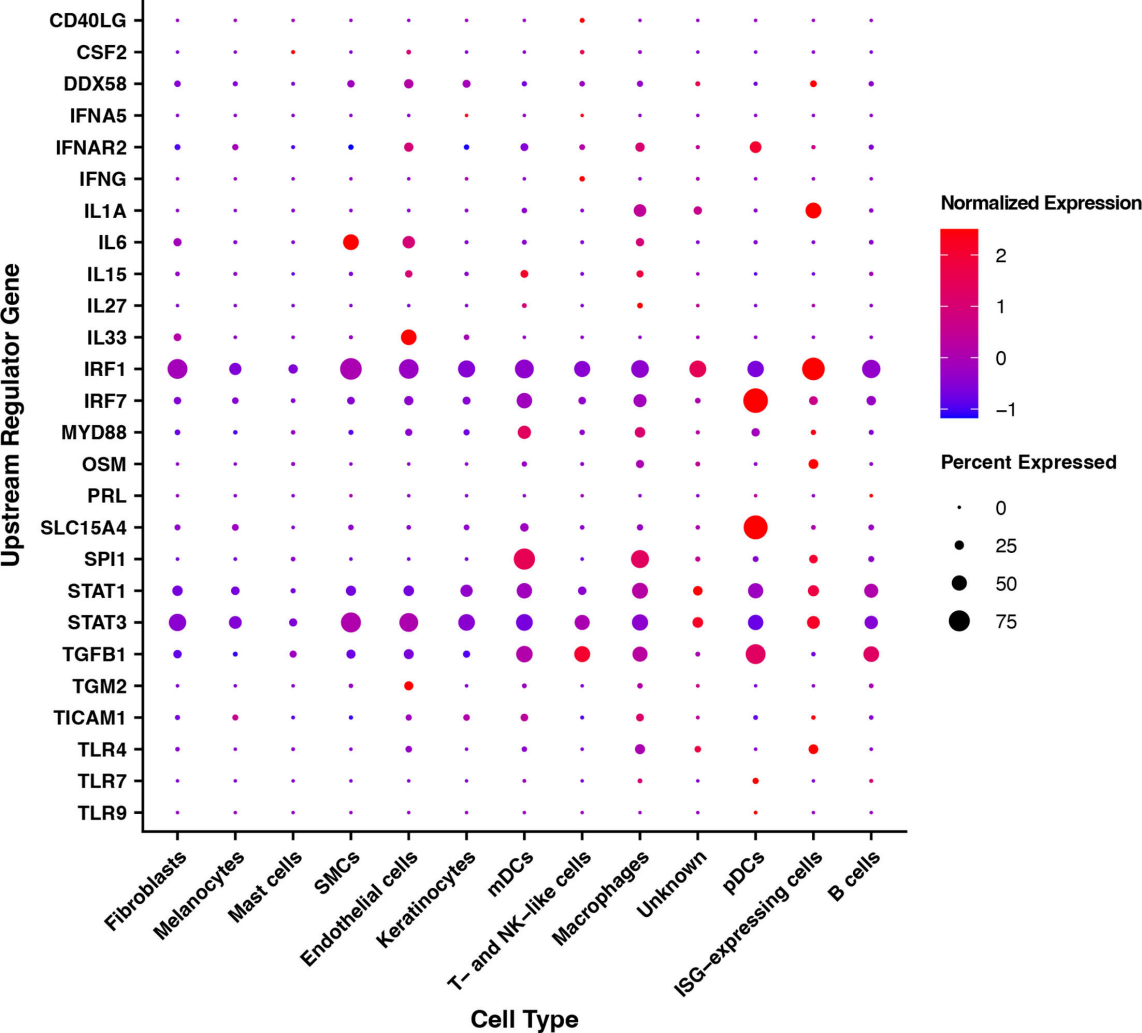
Cell types expressing predicted upstream regulators identified by bulk RNA-seq. Twenty-six of the top 50 predicted upstream regulators were genes that were identified by bulk transcriptomics as being differentially regulated in pustules vs wounds (20) and that were expressed in the scRNA-seq data set. The 26 genes were queried for their differential regulation in the major cell types. Expression is evaluated both on the percentage of cells that expressed the gene (dot size) for that cell type and the normalized expression (e.g., z-score) of the gene compared to the other clusters (dot color).

We previously used bulk RNA-seq to identify DEGs in pustules and wounds (20). We performed Predicted Upstream Regulator Analysis on the top 2,000 DEGs to identify potential molecules and signaling pathways driving the differential gene expression (20). We next asked if a particular cell type was responsible for the enrichment of any of the top 50 predicted upstream regulators. Of the top 50 predicted upstream regulators, 26 were genes that were detected within our scRNA-seq data set (Fig. 2). Several predicted upstream regulators are classically associated with immune activation, such as interferon pathways or endosomal Toll-like receptors (TLRs), and are discussed below.

In the bulk RNA-seq data (20), there were DEGs associated with both pro- and anti-inflammatory responses. In response to interferon stimulation, immunosuppressive programs are typically driven by STAT3 activation, whereas pro-inflammatory programs are typically driven by STAT1 activation (24). On average, most cell types expressed *STAT3*; *STAT1* expression was found primarily in mDCs, pDCs, macrophages, interferon-stimulated gene (ISG)-expressing cells, and B cells (Fig. S2). The highest mean expression of both *STAT1* and *STAT3* was in ISG-expressing cells (Fig. 2). At the single cell level, 14.8% of ISG-expressing cells were *STAT1^+^STAT3^+^*, while 18% were *STAT1^+^* and 25.2% were *STAT3^+^*. Interferon response factor 1 (IRF1), which can complex with STAT1 to drive further *IRF1* and *STAT1* expression among other ISGs (25), was expressed by most cell types but was most highly expressed in ISG-expressing cells and the unknown cell cluster (Fig. 2). These data suggest that, even within the ISG-expressing cell cluster, there is expression of both pro- and anti-inflammatory gene signatures within the site of infection.

TLR7 and TLR9 signaling are predicted to be upregulated in pustules by bulk RNA-seq (20). While the percentage of cells expressing either of *TLR7* or *TLR9* was low (Fig. 2), pDCs had the highest level of *TLR7* and *TLR9* expression. *H. ducreyi* is primarily extracellular in pustules and resists uptake by macrophages and neutrophils, but DCs can ingest *H. ducreyi*, which likely results in activation of these endosomal TLRs (16). Transcription of the histidine/proton transporter *SLC15A4* was also highly upregulated almost exclusively in pDCs. SLC15A4 is an endosomal protein that performs an essential scaffolding function by binding TASL, which can then bind TLR7 and TLR9 and activate transcription of downstream IFN-⍺/β pathway members (26, 27). pDCs also upregulated transcription of the type I interferon receptor (*IFNAR2*), further suggesting that type I interferon plays a role in pustule formation. As specific single nucleotide polymorphisms in *TLR9* are associated with pustule formation versus disease resolution (28), the data suggest that disease outcome may be partially driven by pDCs.

We previously reported that there were 2.8-fold more mDCs than pDCs in pustules (16). Here, the ratio of mDCs to pDCs in pustules was 2.61 ± 0.87 to 1 (mean ± standard deviation), which is congruent with our previous observations (Fig. S1B). Interestingly, there were ~10-fold more pDCs in pustules compared to wounds. In wounds, the mDC:pDC ratio was 24.63 ± 20.32 to 1, which was significantly greater than the mDC:pDC ratio in pustules (*P* = 0.008). pDCs expressed more *CD68* scavenger receptor transcripts than mDCs (Fig. S2). However, mDCs contained more transcripts of pro-inflammatory cytokines, such as *IL1B* and *IL8*, and the T cell co-stimulatory molecules *CD80* and *CD86*, whereas pDCs expressed more *TGFB1* and lacked *CD80* and *CD86* (Fig. S2). Thus, although pDCs are sources of endosomal TLR and type I interferon signaling transcripts (Fig. 2), they also have an immunosuppressive profile, which is consistent with the pustule exhibiting a mixed hyperinflammatory and regulatory state (29).

### Pustules contain mainly CD4^+^ T cells

**Fig 3:**
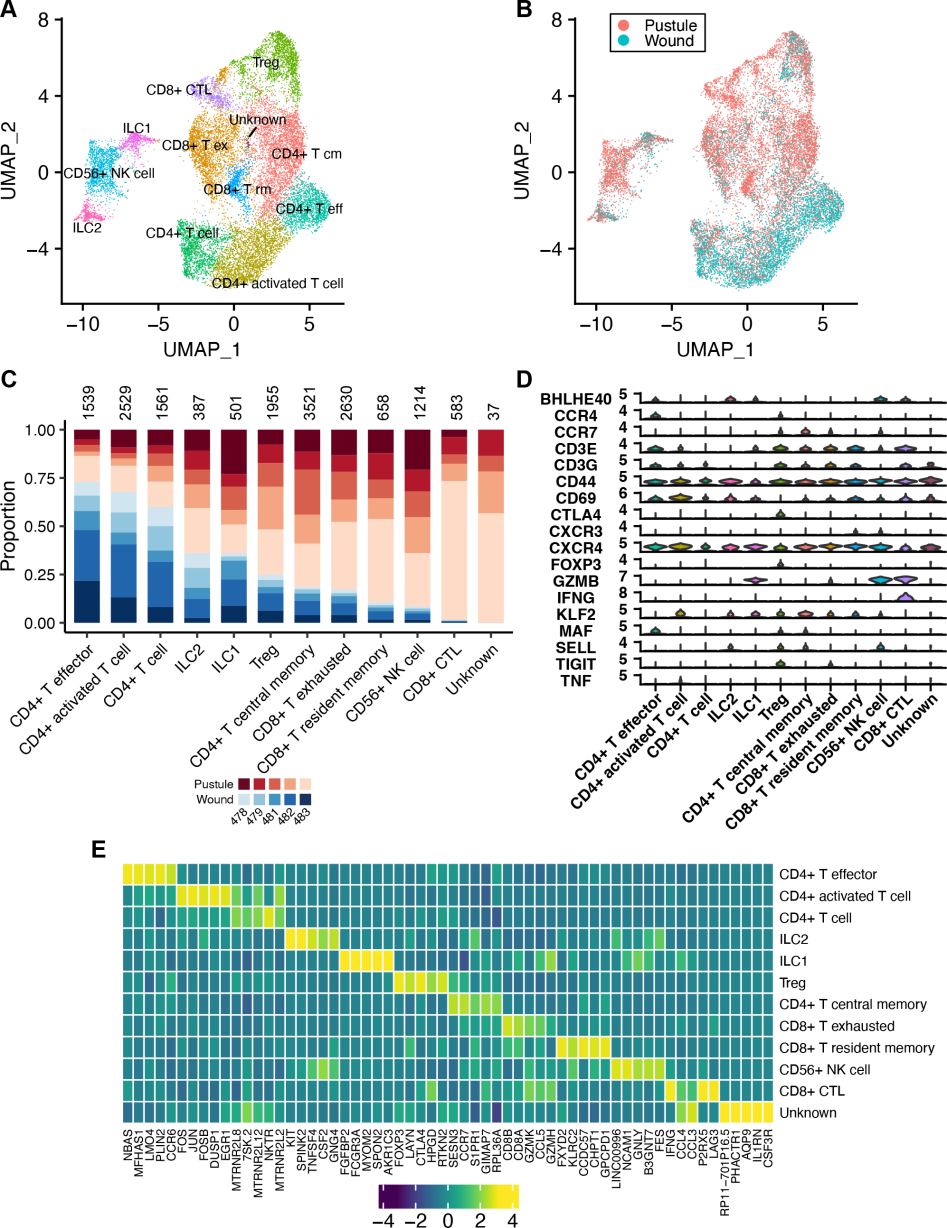
T- and NK-like cell subsets in pustules and wounds. (**A**) UMAP of the 12 T- and NK-like cell subclusters that were identified. (**B**) Dimensional reduction plot showing whether an individual cell was derived from a pustule or wound. (**C**) For each cell type in panel **B**, the proportion of cells derived from each volunteer is shown. Blue colors indicate cells that were derived from a wound; red colors indicate cells that were derived from a pustule. The different shades of colors indicate the volunteer number. The total number of cells belonging to a subcluster is shown above each bar. (**D**) Violin plot showing markers of T and/or NK cells that were used to identify the cell subtypes in panel **A**. (**E**) Heatmap showing the average expression of the top 5 genes in each of the major cell types identified in panel **A**. Gene expression was normalized across subclusters and colored by z-score.

We had previously shown that failure to clear infection was associated with a combined Th1/Treg response (29). We therefore subclustered the T- and NK-like cells to determine the T and NK cell subsets that were present in pustules. We identified 12 subclusters of T- and NK-like cells (Fig. 3A), which included several types of CD4^+^ T cells, CD8^+^ T cells, Tregs, NK cells, and innate lymphoid cells (ILCs). Almost all subsets—CD4^+^ T effector, CD4^+^ activated T cell, CD4^+^ T cell, Treg, CD4^+^ T central memory, CD8^+^ T exhausted, CD56^+^ NK cell, CD8^+^ CTL, and a subcluster with an unknown identity—were significantly differentially abundant in either pustules or wounds (*P* < 0.05). In general, while both pustules and wounds contained various CD4^+^ T cell subsets, pustules contained the majority of CD8^+^ T cells (Fig. 3B; Fig. S3). CD4^+^ T central memory cells were also expanded in pustules compared to wounds (Fig. 3C). The ratio of CD4^+^ to CD8^+^ T cells in pustules was 1.9:1, which is similar to what we had previously detected by flow cytometry and immunohistochemistry (i.e., 2.3- to 3-fold) (15, 17, 30). Additional genes distinguishing the clusters are shown in Fig. 3D and E.

Most T cell subsets expressed *CD44* and *CD69*, indicating that many T- and NK-like cells had been activated (Fig. 3D). In addition, ILC1, CD8^+^ T exhausted, CD56^+^ NK cells, and CD8^+^ CTLs all expressed granzyme B (*GZMB*) transcripts, another marker of activation. Some clusters (ILC2, Treg, CD4^+^ central memory, and CD56^+^ NK cells) expressed L-selectin (*SELL*) transcripts. L-selectin is highly expressed in some T cell subsets including Tregs (31) and can bind to cells expressing the peripheral node addressin carbohydrate epitope present on several proteins involved in cellular adhesion. In addition to *SELL*, Treg, CD4^+^ central memory, and CD56^+^ NK cells also expressed *CCR7*, suggesting that these cells were preparing to migrate to lymph nodes (32).

IFN-γ is an inflammatory cytokine that can be produced by T and NK cells. We previously reported that *H. ducreyi*-specific CD4^+^ cell lines derived from pustules were capable of secreting IFN-γ and IL-10 (33). In contrast, NK cells isolated from endpoint pustules were not capable of secreting IFN-γ (18). Here, *IFNG* was nearly exclusively expressed by CD8^+^ CTL cells (Fig. 3D), suggesting that CD8^+^ CTLs are major producers of IFN-γ in endpoint pustules.

### Infection induces an M1 macrophage response

**Fig 4:**
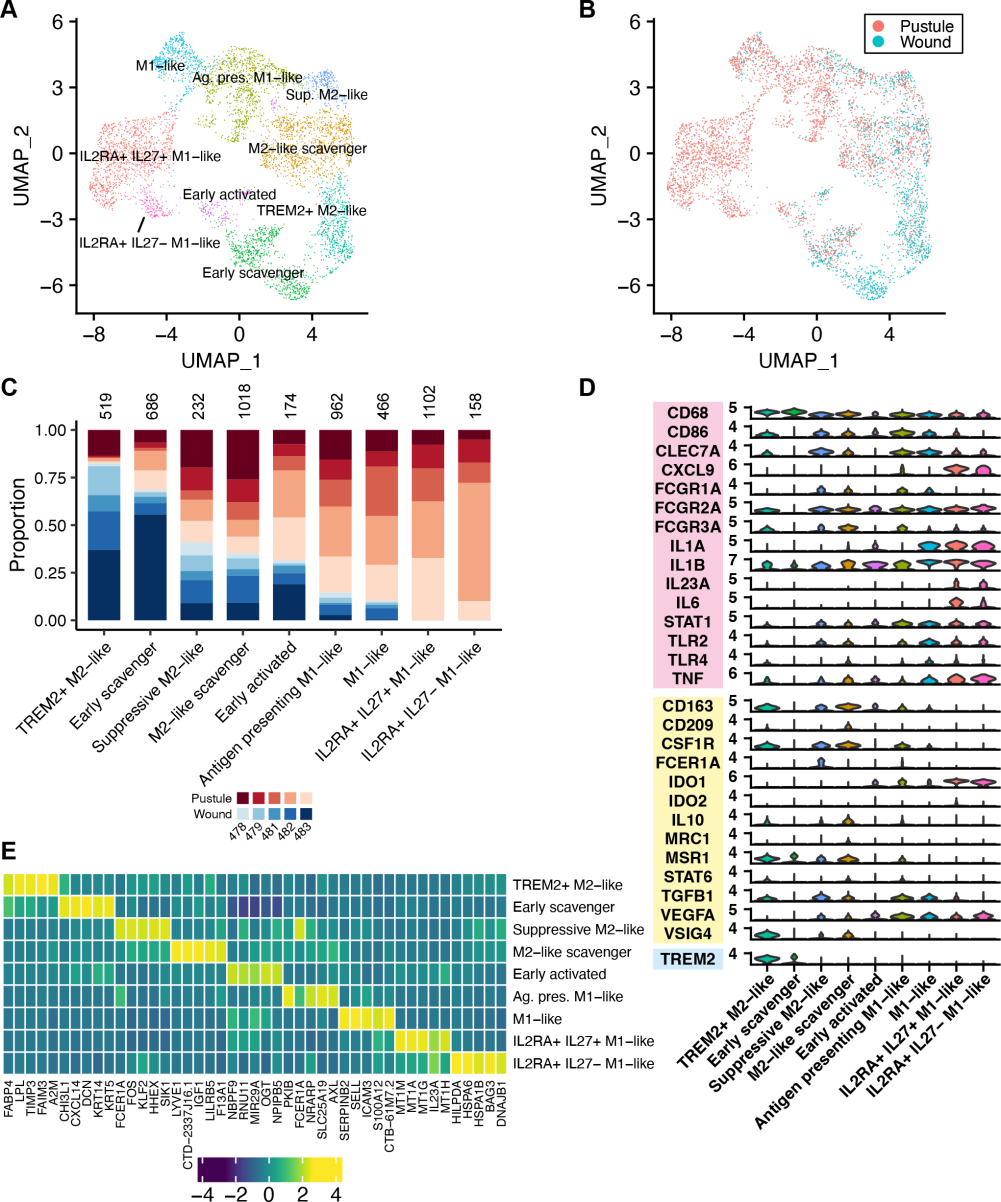
Macrophage subsets in pustules and wounds. (**A**) UMAP of the nine macrophage subclusters. (**B**) Dimensional reduction plot showing whether an individual cell was derived from a pustule or wound sample. (**C**) For each cell type in panel **B**, the proportion of cells derived from each volunteer is shown. Blue colors indicate cells that were derived from a wound; red colors indicate cells that were derived from a pustule. The different shades of colors indicate the volunteer number. The total number of cells belonging to a subcluster is shown above each bar. (**D**) Violin plot showing markers of M1 (pink box) and M2 (yellow box) macrophage subtypes that were used to identify the cell subtypes in panel **A**. *TREM2* (blue box) is a marker of an important macrophage subtype in lepromatous lesions (35). (**E**) Heatmap showing the average expression of the top 5 genes in each of the major cell types identified in panel **A**. Gene expression was normalized across subclusters and colored by z-score.

Macrophages isolated from experimental pustules contain both M1 and M2 markers by flow cytometry (34). We identified nine macrophage subclusters by scRNA-seq (Fig. 4A). Wounded sites contained more M2-like macrophages while pustules contained more M1-like macrophages (Fig. 4B and C). In concordance with previous flow cytometry data (34), we did not identify purely canonical M1 or M2 macrophages, since most cells expressed both M1 and M2 markers (Fig. 4D). DEGs for each cluster are shown in Fig. 4E.

There were several types of macrophages that were differentially abundant in pustules compared to wounds: TREM2^+^ M2-like, Early scavenger, M1-like, IL2RA^+^ IL27^+^ M1-like, and IL2RA^+^ IL27^−^ M1-like (*P* < 0.05); the latter three M1 types were enriched in pustules. The M1-like cluster expressed major histocompatibility complex (MHC) class II transcripts, *CD86, SELL*, and *ICAM3* (Fig. 4D and E; Fig. S4A), indicating that these macrophages could present antigen to CD4^+^ T cells. Interestingly, all but the Early scavenger macrophages expressed at least one activating Fcγ-receptor (i.e., *FCGR1A*, *FCGR2A*, and *FCGR3A*) (Fig. 4D), indicating that these macrophages had the potential to phagocytose opsonized particles (36). Signal activation through Fcγ-receptors requires IgG bound to antigen to cross-link them together. As volunteers experimentally infected to the pustular stage of disease do not have detectable *H. ducreyi-*specific IgG in sera obtained during or after infection (15, 37), any cross-linking IgG, if present, is unlikely to be *H. ducreyi*-specific. Even if such cross-linking occurred, *H. ducreyi* prevents FcγR-mediated phagocytosis by inactivation of Src family kinases during infection by secreting the anti-phagocytic effectors LspA1 and LspA2 (14, 38).

Two macrophage clusters were found exclusively in pustules: IL2RA^+^ IL27^+^ M1-like and IL2RA^+^ IL27^−^ M1-like (Fig. 4C). Both macrophage clusters expressed pro-inflammatory cytokines (e.g., *IL23A* and *IL6*) and had high expression of the anti-inflammatory gene *IDO1. H. ducreyi* lipooligosaccharide (LOS) induces IDO1 expression in DCs (39); we hypothesize that a similar LOS-dependent mechanism induces *IDO1* expression in these macrophages. Both clusters also expressed *IL2RA*, which, when bound by IL-2, activates pro-inflammatory pathways (40, 41). While all macrophage subtypes expressed minimal levels of the *IL2RB* subunit, the common γ-chain (*IL2RG*) was expressed by multiple macrophage clusters including the IL2RA^+^ cells (Fig. S4A). The larger cluster of the two IL2RA^+^ macrophages also expressed transcripts for both subunits of IL-27: *IL27* and *EBI3* (Fig. S4A). IL-27 increases T cell survival and recruitment via IL-15 in skin allergy (42). Macrophages that expressed *IL27* also expressed *IL15* and the highest levels of metallothionein transcripts (Fig. S4A), which encode cysteine-rich proteins that can bind metals in the environment to combat oxidative stress. The IL-2RA^+^ IL-27^−^ macrophages expressed many heat shock proteins, indicating that these cells were responding to protein-folding induced stress during infection (Data set S1).

The two IL2RA^+^ M1-like cell clusters could also be differentiated from the other macrophage cell types by their expression of matrix metalloproteinase 14 (MMP-14) transcripts compared to the other subtypes that expressed *MMP9* (Fig. S4A). MMP-14 cleaves proMMPs—including proMMP-9. Chronic, non-healing diabetic foot ulcers and pressure ulcers are associated with higher levels of *MMP9* and/or *MMP14* than healed ulcers (43, 44). Whether the *MMP9* and/or *MMP14* expression observed here produces an amount of basement membrane proteolysis that improves immune cell migration through the tissue or leads to the excessive production of damage-associated molecular patterns (DAMPs) is unknown. We hypothesize that the IL2RA^+^ M1-like clusters make up the macrophage collar that surrounds the abscess (13, 14), since they express transcripts corresponding to *IDO1*, anti-oxidative defenses, and MMPs. This environment may in turn drive the differentiation of FOXP3^+^ Tregs within the abscess collar.

### Infection induces an interferon response

**Fig 5:**
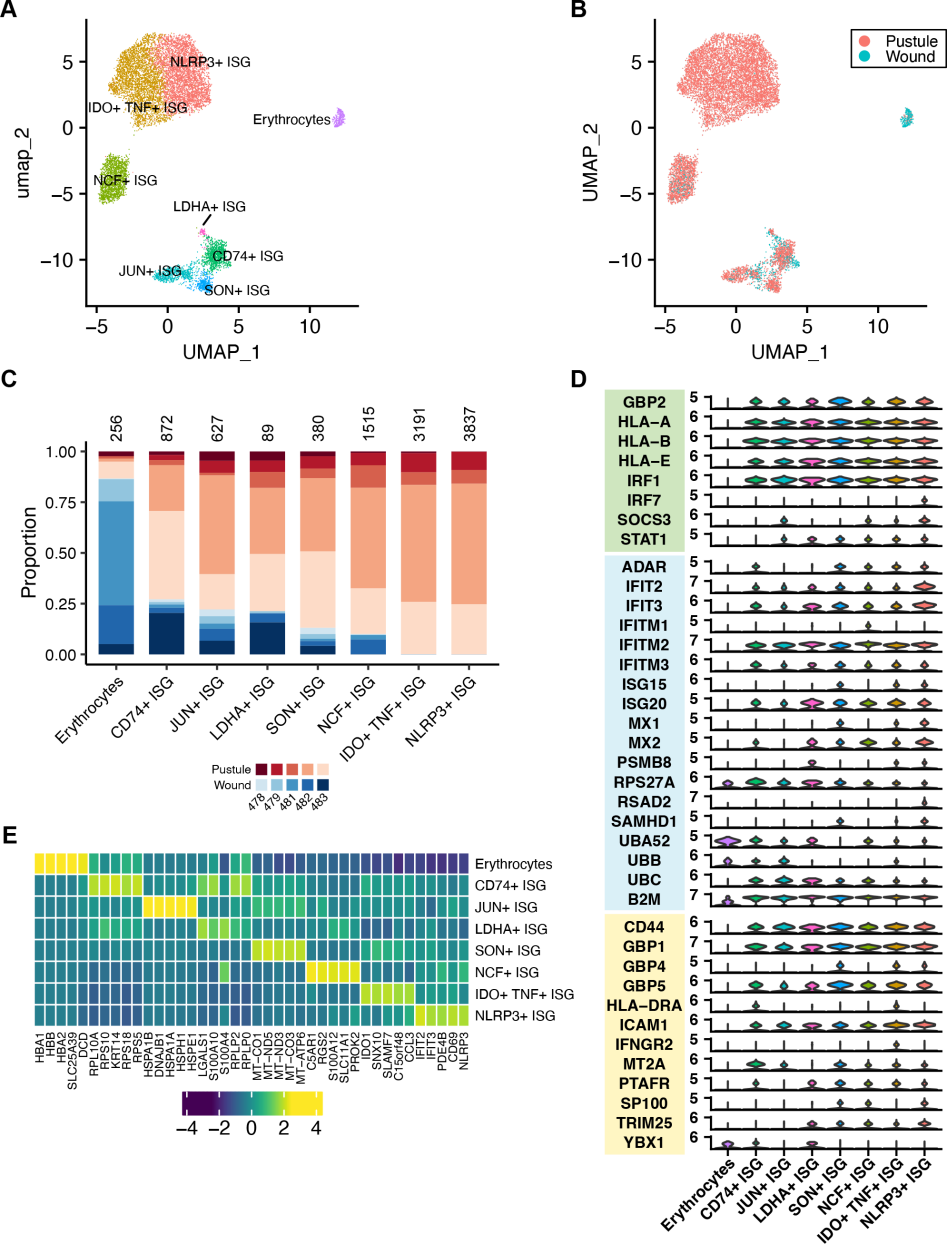
ISG-expressing cells in pustules and wounds. (**A**) Eight subclusters of ISG-expressing cells were identified. One cluster displayed high expression of hemoglobin genes and was identified to be erythrocytes. (**B**) Dimensional reduction plot showing whether an individual cell was derived from a pustule or wound sample. (**C**) For each cell type in panel **A**, the proportion of cells derived from each volunteer is shown. Blue colors indicate cells that were derived from a wound; red colors indicate cells that were derived from a pustule. The different shades of colors indicate the volunteer number. The total number of cells belonging to a subcluster is shown above each bar. (**D**) Violin plot showing the expression of interferon-stimulated genes. Gene sets for ISGs related to IFN-⍺/β and IFN-γ pathways were downloaded from the Reactome database. ISGs with median expression >1 in at least one cluster are shown. ISGs that are expressed when stimulated by either IFN-⍺/β and IFN-γ stimulation (green box) or that are unique to IFN-⍺/β stimulation (blue box) or IFN-γ stimulation (yellow box) are shown. (**E**) Heatmap showing the average expression of the top 5 genes in each of the major cell types identified in panel **A**. Gene expression was normalized across subclusters and colored by z-score.

We identified a cluster of cells that expressed a high number of ISGs that was composed of eight subclusters (Fig. 5A). Most ISG-expressing cells were in pustules (Fig. 5B and C), but only the IDO^+^ TNF^+^ ISG-expressing and NLRP3^+^ ISG-expressing cells were significantly differently abundant between pustule and wound samples (*P* < 0.05). One subcluster was comprised of erythrocytes that expressed high levels of hemoglobin genes (*HBA1*, *HBA2*, and *HBB*) (Fig. 5E; Data set S1); all others expressed *CSF3R* and *FCGR2A*, various class I major histocompatibility complex genes (*HLA-A*, *HLA-B*, and *HLA-E*), and antigen presentation markers such as *CD69* and *CD14*, suggesting that the cells were professional antigen-presenting cells of monocytic origin (Fig. 5D and E; Fig. S4B and C). Hereafter, when we refer to “ISG-expressing cells,” we exclude the erythrocyte cluster.

All ISG-expressing cells expressed the pro-inflammatory cytokine genes *IL1A* and *IL1B* (Fig. S4B). Macrophages are a major source of IL-1, and IL-1 skews macrophages toward an M1 phenotype (45). ISG-expressing cells all primarily expressed canonical M1 markers with the notable exception of IDO^+^ TNF^+^ ISG-expressing cells, which also expressed the M2 marker *IDO1* (Fig. 5E; Fig. S4B). IL-1α and IL-1β production can be stimulated via superoxide or inflammasome activation via IRFs. The ISG-expressing cells expressed the highest levels of *IRF1* (Fig. 2), which opens chromatin at interferon-stimulated response element sites and allows other IRFs to bind and stimulate IFN-α and IFN-β production. All ISG subclusters expressed *IRF1*, and the NLRP3^+^ ISG-expressing cell subcluster also expressed *IRF7* (Fig. 5D). We previously showed that *H. ducreyi* infection of monocyte-derived macrophages induces NLRP3-dependent production of IL-1β (46). Despite the ubiquitous expression of *IL1B* across subclusters, *NLRP3* expression was confined to one subset of ISG-expressing cells (i.e., the NLRP3^+^ ISGs) (Fig. 5E; Fig. S5C).

Using the IFN-⍺/β and IFN-γ response gene sets from the Reactome database (47), we examined the expression of ISGs in each subcluster that are expected to be regulated by both IFN-⍺/β and IFN-γ stimulation, IFN-⍺/β stimulation alone, or IFN-γ stimulation alone (Fig. 5D). Although the expression of IFN-⍺/β-stimulated genes occurred in all ISG clusters, the expression of the receptors for IFN-⍺/β, *IFNAR1* and *IFNAR2*, was extremely low for all cells (Fig. S4C), suggesting that these cells already expressed these receptors. *IFNGR2* was expressed in the IDO^+^ TNF^+^ ISG cluster (Fig. S4C). However, the expression of *IDO1* in the IDO^+^ TNF^+^ ISG subcluster was pustule-specific, suggesting that active IFN-γ-dependent signaling occurred during infection.

The remaining ISG-expressing cell clusters had distinct phenotypes (Fig. 5D and E; Fig. S4B and C; Data set S1). For example, lactose dehydrogenase (*LDHA*) was exclusively found in the LDHA^+^ ISGs (Fig. S4C), suggesting that these cells were using fermentation to generate energy. The LDHA^+^ ISG and CD74^+^ ISG clusters both expressed the IFN-γ-inducible transcription factor *YBX1*, which increases translation (Fig. S4C) (48, 49). Both clusters had a high abundance of ribosomal protein transcripts as well as other regulatory proteins for translation (e.g., *EEF1B2*) (Fig. 5E; Fig. S4C). NCF^+^ ISGs expressed *NCF1* and *NCF2*, which are needed to activate superoxide production by NADPH oxidase, as well as *TLR4* and *CD14*, suggesting that these cells could respond to *H. ducreyi* LOS and were producing superoxide (Fig. S4B and C). This is consistent with the upregulation of TLR4 signaling pathway members in pustule formers (29). The NCF^+^ ISG cells also expressed complement 5a receptor (*C5AR1*) (Fig. 5E), which has been associated with pro-inflammatory responses in macrophages, and lysozyme (*LYZ*) transcripts (Fig. S4C).

### Inflammatory signals in the microenvironment

Immune cells are recruited to sites of infection where they can sense DAMPs and/or pathogen-associated molecular patterns. Although *H. ducreyi* does not co-localize with keratinocytes in pustules (14), lesional keratinocytes are swollen (14), indicating that infection does affect them. Similarly, *H. ducreyi* does not co-localize with fibroblasts in pustules, but it does bind to newly secreted collagens I and III secreted by fibroblasts at the base of the pustule (14). We showed that endothelial cells in pustules express E-selectin (*SELE*); E-selectin aids in trafficking of CLA^+^ T cells from the blood stream into the tissue (17). As the contribution of stromal cells in *H. ducreyi* infection has not been examined in detail, we next compared the transcriptional responses of keratinocytes, fibroblasts, and endothelial cells in pustules and wounds.

**Fig 6:**
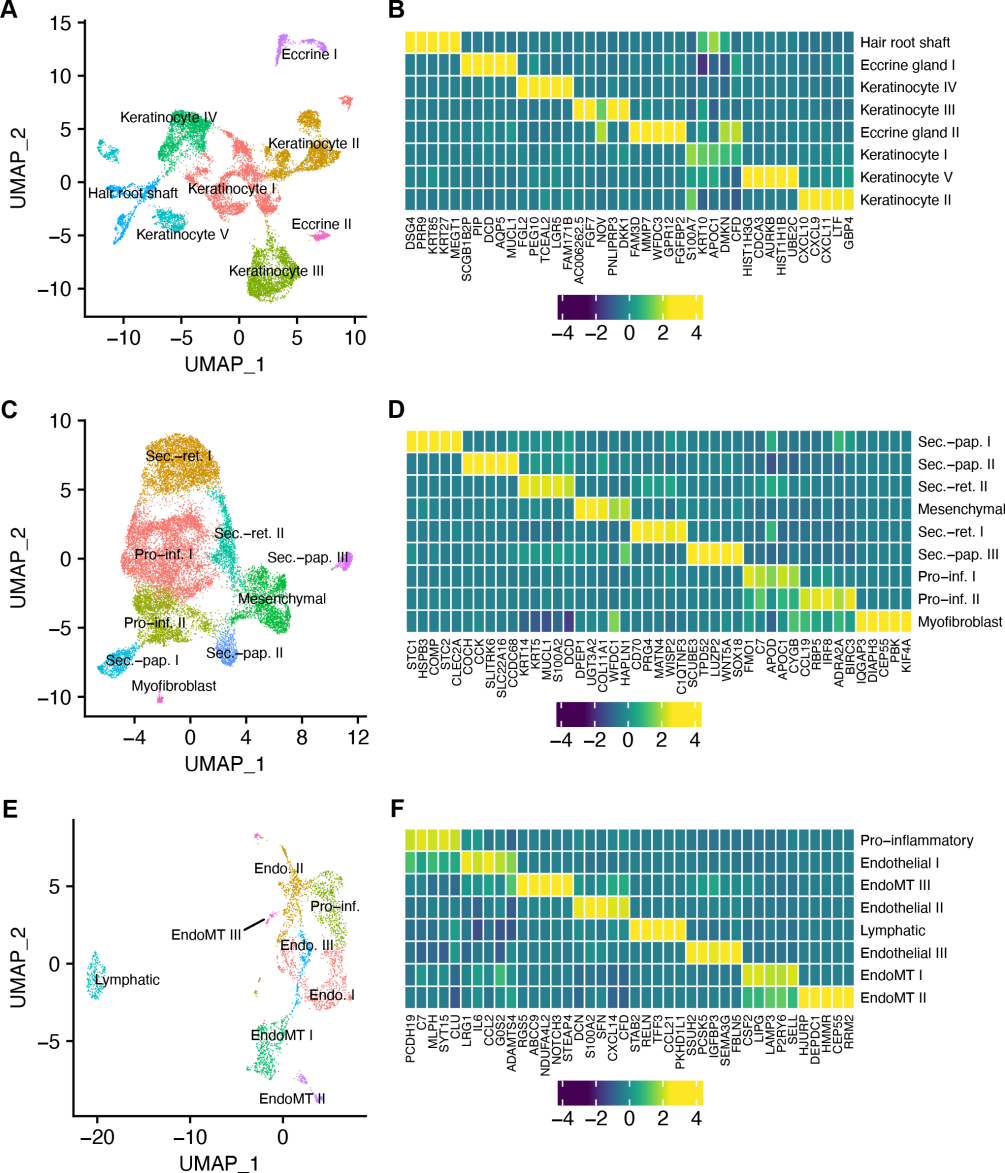
Types of resident skin cell in pustules and wounds. (**A**) UMAP of the eight subclusters of keratinocytes. (**B**) Heatmap showing the average expression of the top 5 genes in each of the keratinocyte subclusters identified in panel **A**. (**C**) UMAP of the nine subclusters of fibroblasts. (**D**) Heatmap showing the average expression of the top 5 genes in each of the fibroblast subclusters identified in panel **C**. (**E**) UMAP of the eight subclusters of endothelial cells. (**F**) Heatmap showing the average expression of the top 5 genes in each of the endothelial cell subclusters identified in panel **E**. For panels **B**, **D**, and **F**, gene expression was normalized across subclusters and colored by z-score.

We identified eight subclusters of keratinocytes (Fig. 6A). The top 5 differentially expressed genes between keratinocyte clusters are shown in Fig. 6B; the relative abundances of the different cells and additional genes are in Fig. S5. There were three subclusters of specialized keratinocytes: one cluster of hair root shaft keratinocytes and two types of eccrine gland cells. The remaining five clusters were typical keratinocytes. None of the keratinocyte subclusters were differentially abundant between pustules and wounds (*P* > 0.05), but keratinocyte II cells trended toward being more abundant in pustules (*P* = 0.06). Keratinocyte II cells were the only keratinocytes that expressed chemokines such as *CXCL9*, *CXCL10*, and *CXCL11* and ISGs such as *ISG15*, *MX1*, and *WARS*, suggesting that these cells were stimulated by both IFN-⍺/β and IFN-γ at infected sites.

The keratinocyte I–V clusters all expressed *KRT5* and *KRT14*, indicating that these were all basal keratinocytes. However, they also expressed *KRT10*, indicating that the cells were differentiating. This dysregulated keratin expression pattern is commonly seen in stressed keratinocytes (50), and combinations of stress keratins (*KRT6A*, *KRT6B*, *KRT16*, and/or *KRT17*) were expressed in the keratinocyte I–V clusters (Fig. S5).

We identified nine fibroblast subclusters (Fig. 6C). There were three types of secretory-papillar fibroblasts, two types of secretory-reticular fibroblasts, two types of pro-inflammatory fibroblasts, mesenchymal fibroblasts, and myofibroblasts (Fig. 6C). The top 5 differentially expressed genes among fibroblasts are shown in Fig. 6D. Their relative abundance and additional genes are shown in Fig. S6. Fibroblast types were similarly abundant in pustules and wounds, except for secretory-papillary I fibroblasts, which were higher in wounds, and myofibroblasts, which were higher in pustules (*P* < 0.05). Both myofibroblasts and mesenchymal fibroblasts expressed *POSTN*, indicating that they were producing collagen (Fig. S6B) (51). In addition, the myofibroblasts also expressed *ENG* (Fig. S6B), which is a membrane receptor that binds TGF-β receptors on other cells and is associated with the induction of fibrosis (52). Hypertrophic scar formation occurs in 16.8% (95% CI: 12.5, 21.2) of 285 persons who underwent biopsies of pustules (12; S. M. Spinola, unpublished data), suggesting that activation of myofibroblasts may be responsible for this adverse event. Conversely, the secretory-papillary II and III, secretory-reticular I, pro-inflammatory I and II, and myofibroblast clusters expressed *FGF2* (Fig. S6B), which inhibits fibrosis. Of note, the pro-inflammatory I cluster cells expressed 9 out of the 10 marker genes of the fibroblast I cells described in Wiedemann et al., which participate in immune surveillance and response in normal skin (53).

We identified eight subclusters of endothelial cells (Fig. 6E). The top 5 differentially expressed genes are shown in Fig. 6F; their relative abundance and select additional transcripts are shown in Fig. S7. All endothelial cells expressed *ENO1*, *VIM*, and *FABP4* (Fig. S7B). Lymphatic endothelial cells expressed the canonical markers *PROX1*, *FLT4*, and *LYVE1*; the remaining seven clusters were blood endothelial cells (e.g., expressed *FLT1*). The seven blood endothelial cell clusters contained a pro-inflammatory cluster, three clusters that had markers related to the endothelial-mesenchymal transition (EndoMT) such as *TAGLN*, and three clusters that expressed traditional endothelial cell markers. The pro-inflammatory, endothelial I, EndoMT III, and lymphatic cells were significantly more abundant in wounds; the EndoMT I and II clusters were significantly more abundant in pustules (*P* < 0.05). EndoMT I and II expressed high levels of *SERPINE1*, which is involved in healing vascular ruptures by breaking blood clots and stimulating cell migration. They also expressed *SELE* (Fig. S7), which is highly expressed in pustules and is involved in trafficking of CLA+ CD4 T cells into infected sites from capillaries (17). Several genes also suggest that the NF-𝜅B pathway is active in most endothelial cell clusters—including EndoMT I and II—such as *TIFA* and *IL33* (Fig. S7B).

Experimental abscesses that form from subcutaneous injection of bacteria are hypoxic (54), and *H. ducreyi* can respond to and survive in anaerobic environments (55). Except for the endothelial II cluster, all endothelial clusters expressed the hypoxia transcription factor *HIF1A* (Fig. S7B). The EndoMT I and II, pro-inflammatory, and endothelial I clusters also expressed *ENTPD*, which is expressed during ischemia reperfusion (Fig. S7B). Expression of *HIF1A* and *ENTPD* support that the sites of injury and infection are hypoxic.

### TGF-β, IL-1β, and TNF-α signaling in human skin

TGF-β transcripts are highly expressed in lesions of reinfected pustule formers (29). To identify additional potential cellular sources of TGF-β and IL-1β and the cells that were potentially stimulated by these cytokines, we performed receptor-ligand analysis using CellPhoneDB (56, 57). *TGFB1* transcripts from all immune cell types except mast cells significantly interacted with TGF-β receptor transcripts on keratinocytes (and other stromal cell types) (Fig. S8A). Keratinocytes I–V and the hair root shaft cluster also expressed *ITGAV* (Fig. S5B), which encodes the α-subunit of integrin ⍺_V_β_6_ and activates TGF-β bound to the extracellular matrix (58). High TGF-β expression in the epidermis coupled with high expression of ⍺_V_β_6_ is associated with chronic wound states (59, 60). TGF-β is important for cross-talk between fibroblasts and keratinocytes during wound closure and can lead to the differentiation of fibroblasts into myofibroblasts (61, 62).

Keratinocytes were the primary recipients of IL-1β signaling (Fig. S8B). The cellular sources of IL-1β were macrophages and ISG-expressing cells, which are activated and of monocytic origin. *In vitro*, only non-polarized monocyte-derived macrophages secreted IL-1β in response to *H. ducreyi* (46). Our *in vivo* data predict that both macrophages and ISG-expressing cells secrete IL-1β.

TNF-α can be secreted by keratinocytes in response to bacterial infection, but keratinocytes do not secrete TNF-α in response to *H. ducreyi* infection (63). However, our receptor-ligand analysis suggests that TNF-α signals to fibroblasts, keratinocytes, melanocytes, endothelial cells, and smooth muscle cells (SMCs) and that TNF-α is primarily secreted by macrophages, ISG-expressing cells, and mDCs (Fig. S8C). TNF-α can induce IDO in DCs *in vitro* (39), which is also consistent with our analysis (Fig. S2 and 8C).

### Localization of antigen-presenting cells in tissue

**Fig 7:**
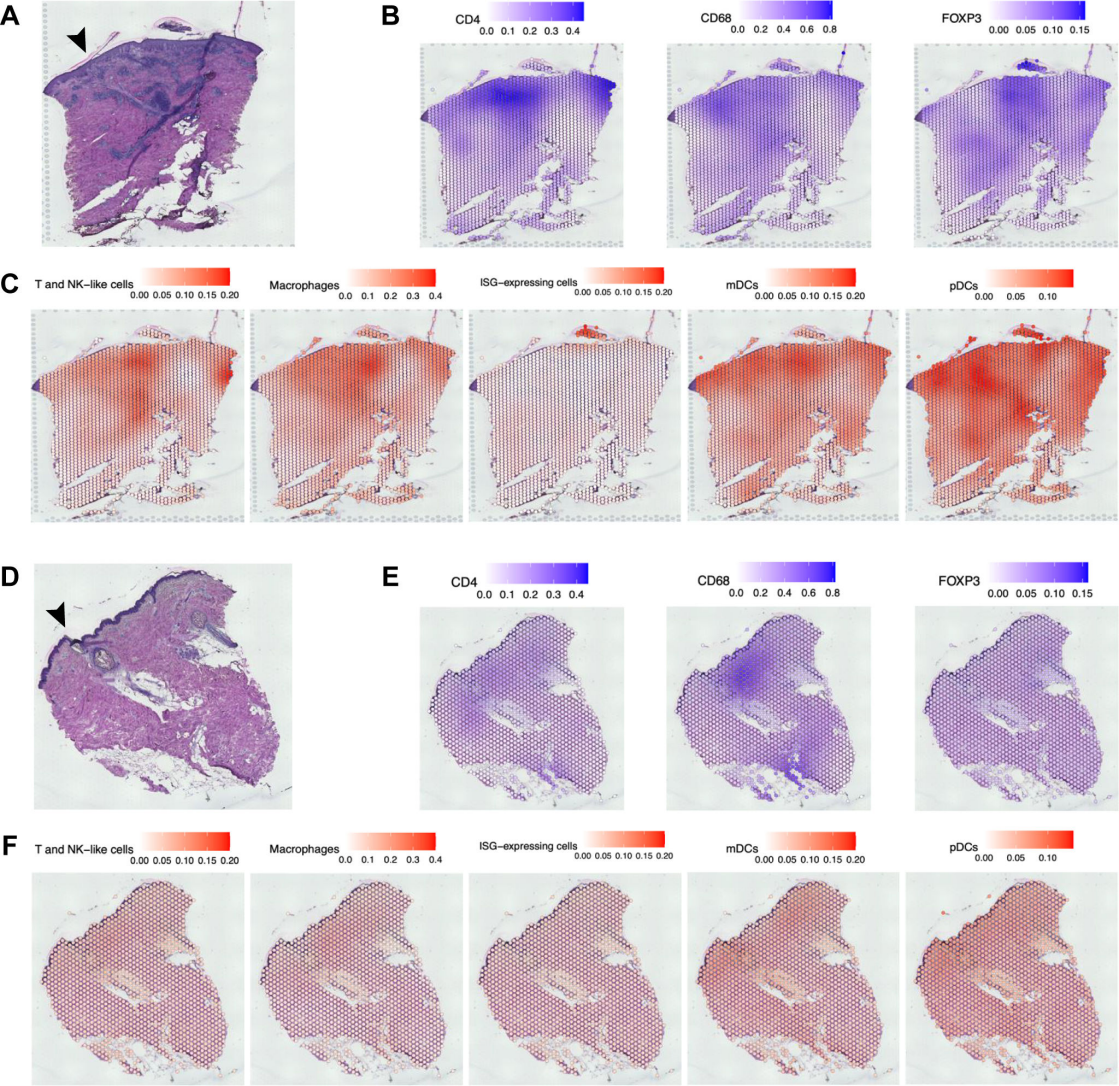
Spatial transcriptomics analysis of pustule and wound samples. (**A**) Representative H&E staining of a pustule from volunteer 479. (**B**) Feature plots for the expression of immune markers *CD4*, *CD68*, and *FOXP3* in panel **A**. (**C**) Feature plots of the spatial distribution of T- and NK-like cells, macrophages, ISG-expressing cells, mDCs, and pDCs in panel **A**. (**D**) Representative H&E staining of a wounded site from volunteer 479. (**E**) Feature plots for the expression of immune markers *CD4*, *CD68*, and *FOXP3* in panel **D**. (**F**) Feature plots of the spatial distribution of T- and NK-like cells, macrophages, ISG-expressing cells, mDCs, and pDCs in panel **D**. Arrows indicate the epidermis.

We performed spatial transcriptomics on pustules and wounds from four of the volunteers on the 10x Genomics Visium platform. Since the Visium platform can only resolve to ~5–10 cells per spot, we deconvolved the spatial transcriptomics data using the scRNA-seq data and SPOTlight, a non-negative least squares (NNLS) regression algorithm (64). Consistent with our previous observations (15), hematoxylin & eosin (H&E) staining of the pustule showed a swollen, inflamed epithelium and dense immune infiltrate (Fig. 7A). Areas that contained immune infiltrate staining were consistent with higher *CD4*, *CD68*, and *FOXP3* expression (Fig. 7B) and the occurrence of T- and NK-like cells, macrophages, ISG-expressing cells, mDCs, and pDCs in the tissue (Fig. 7C). Wounds lacked a noticeable immune infiltrate, and the epithelial thickness was normal (Fig. 7D). Expression of *CD4*, *FOXP3*, and *CD68* was reduced in wounds compared to pustules, although some *CD4* and *CD68* expression was still present (Fig. 7E). Compared to pustules, there was little signal from macrophages, ISG-expressing cells, mDCs, and pDCs in wounds (Fig. 7F). T- and NK-like cells were significantly spatially correlated with macrophages, ISG-expressing cells, mDCs, and pDCs in pustules compared to wounds (*P* < 0.05; Fig. S9; Table S2).

ISG-expressing cells were only significantly spatially associated with keratinocytes (*P* = 0.04; Table S2), which is consistent with their localization at the top of the pustule (Fig. 7C). Keratinocytes were also associated with T- and NK-like cells, multiple antigen-presenting cells (macrophages, pDCs, mast cells, and B cells), and fibroblasts (Table S2). *In vitro, H. ducreyi* is phagocytosed by monocyte-derived mDCs, which can then present antigen (16). *In vivo*, macrophages and pDCs were associated with each other as well as mDCs, keratinocytes, endothelial cells, T- and NK-like cells, and B cells (Table S2). However, mDCs were not associated with the stromal cells (keratinocytes, fibroblasts, or endothelial cells) or B cells. Together, these data suggest that pDCs and macrophages are found together throughout the infected sites, whereas mDCs primarily localize with the immune infiltrate where they may present *H. ducreyi* antigens.

### Systems biology modeling detects elevated antigen presentation signals

**Fig 8:**
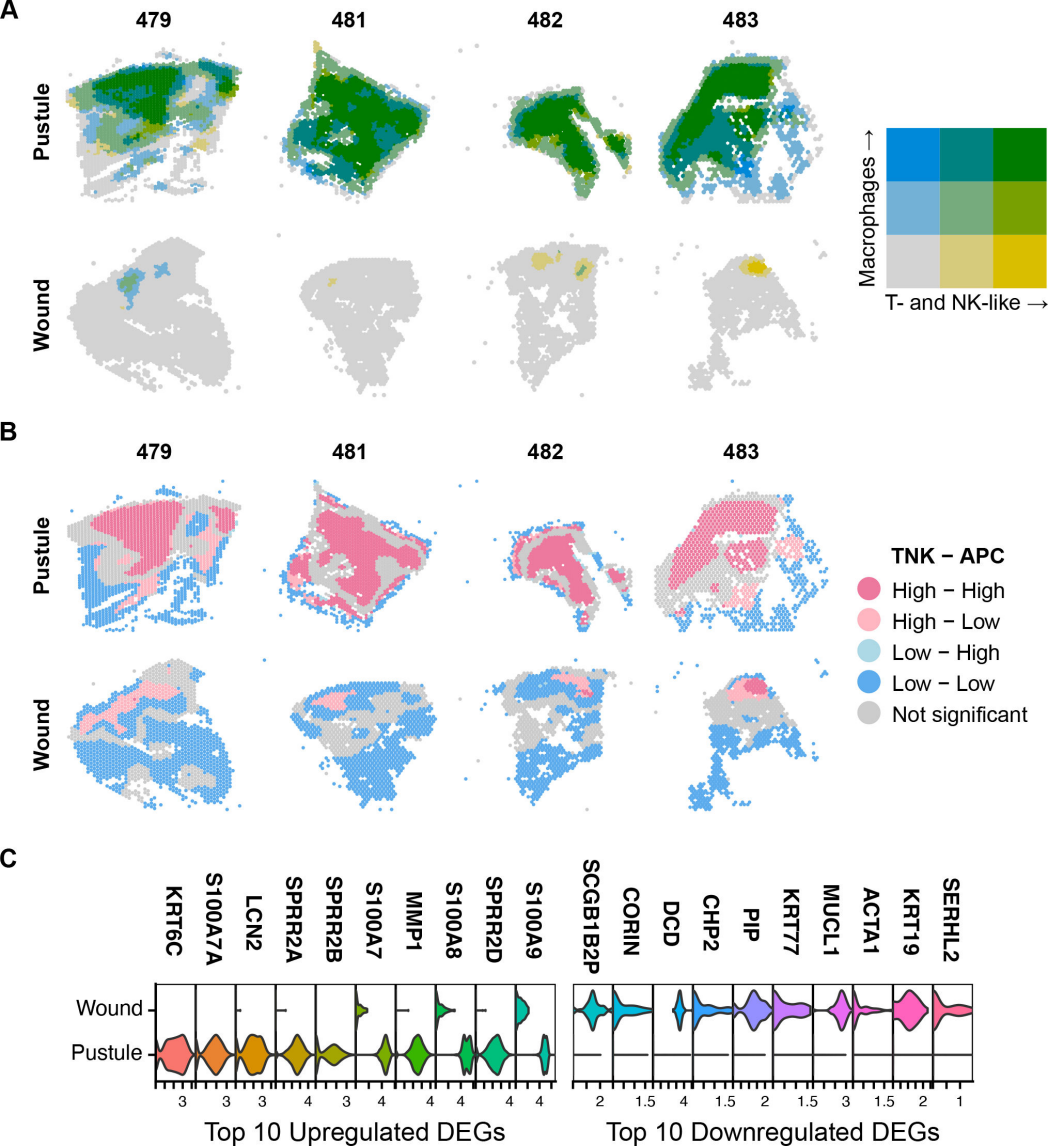
Antigen presentation within pustule and wound sites. (**A**) Bivariate choropleth maps of the co-occurrence of T- and NK-like cells (yellow) and macrophages (blue) in each Visium barcode spot. Gray areas lack any signal from either cell type, dark green indicates high signal from both cell types, while blues or yellows indicate that only one cell type is present. Breaks across colors were normalized across the pustule-wound pair for each volunteer. (**B**) Local indicators of spatial association plot of TNK with the KEGG antigen presentation gene set. (**C**) Differential gene expression analysis in pustules vs wounds for areas with high TNK and antigen presentation cell (APC) signal.

Given the high association of T- and NK-like cells with different antigen presentation cell types (e.g., macrophages, ISG-expressing cells, mDCs, and pDCs) in infected tissue (Fig. 8A; Fig. S10), we further examined antigen presentation within the tissue. To define potential areas of antigen presentation in pustules and wounds, we determined the Moran’s I coefficient between T- and NK-like cells and the presence of the antigen presentation gene module (from Kyoto Encyclopedia of Genes and Genomes [KEGG]) within the tissue. All pustules contained large areas of potential antigen presentation to T- and NK-like cells, while the wounded sites contained few if any areas (Fig. 8B). To gain additional insight into the transcriptional differences between areas of antigen presentation in pustules and wounds, we performed differential gene expression analysis between areas with high T- and NK-like cells and high antigen presentation in pustules versus wounds. Using cutoffs of a log2 fold change >0.5 and an adjusted *P*-value <0.05 to define significance, we identified 92 upregulated genes and 244 downregulated gene areas of antigen presentation in pustules versus wounds (Data set S2). The top 10 upregulated and downregulated genes in pustules compared to wounds are shown in Fig. 8C.

We used gene sets in the gene ontology biological process, Reactome, BioCarta, KEGG, and PID databases to perform pathway analysis on the gene lists. In pustules, genes involved in “T cell chemotaxis,” “regulation of Toll-like receptor signaling pathway,” “antimicrobial peptides,” “IFN-γ pathway,” “IFN-α/β signaling,” and “cellular response to IL-1” were present in areas of antigen presentation. These areas also contained genes involved in “keratinization,” “intermediate filament reorganization,” “gap junction assembly,” and “extracellular disassembly,” suggesting that antigen presentation occurred closer to the epidermis in pustules than in wounds. The presence of these pathways is consistent with the location of areas that are high in T- and NK-like cells and antigen presentation signatures (Fig. 8B) and with the characteristics of the immune infiltrate observed by H&E staining (Fig. 7). In wounds, genes involved in “regulation of fibroblast growth factor receptor signaling pathway,” “extracellular matrix assembly,” “collagen fibril formation,” and “monocyte chemotaxis” were upregulated in the few areas of antigen presentation, suggesting that wound healing is active at those sites.

## DISCUSSION

A major gap in the understanding of infectious diseases is the relative lack of information about molecular interactions between pathogens and the human host. We previously used bulk transcriptomics for *H. ducreyi*-infected skin to broaden our understanding of *H. ducreyi* pathogenesis and provide a framework for the development of therapeutics or vaccines that go beyond investigating the contributions of individual genes of either the pathogen or the host to infection (20, 65–67). However, a limitation of bulk transcriptomics is that cell type information is lost during sample preparation. Here, we overcame this limitation by using scRNA-seq and spatial transcriptomics to characterize host cell subsets within experimentally infected and wounded human skin.

We had previously characterized the cutaneous immune response to *H. ducreyi* by flow cytometry and immunohistochemistry and had identified several major cell types that are recruited to infected sites including neutrophils, mDCs, pDCs, CD4^+^ and CD8^+^ central memory and effector T cells, CD4^+^FOXP3^+^ Treg cells, activated NK cells, and M1 and M2 polarized macrophages (16–18, 34, 68, 69). This study identified multiple subtypes of T- and NK-like cells, ISG-expressing cells, and macrophages in infected sites. pDCs and ISG-expressing cells were major drivers of the differentially expressed genes detected between infected and wounded sites (Fig. 2) (20). We were unable to detect neutrophils by scRNA-seq. This is likely due to neutrophils containing few transcripts and large amounts of RNases. We were also unable to identify any *H. ducreyi* transcripts in our spatial transcriptomics data, which may be due to bacterial transcripts lacking poly-A tails and the overall low numbers of bacteria present in infected tissue (70, 71).

Modlin and co-workers previously used dual RNA-seq on skin biopsies of lesions of patients infected with *Mycobacterium leprae,* an intracellular pathogen of macrophages that causes a chronic infection that can be self-contained and paucibacillary (called tuberculoid leprosy [T-lep] or reversal reactions [RR]) or not contained and multibacillary (called lepromatous leprosy [L-lep]) (72). In comparing L-lep to T-lep lesions, they found that the expression of bacterial transcripts correlates with bacterial burden and a host type I interferon gene signature (72). Using scRNA-seq and spatial transcriptomics of biopsies of RR and L-lep lesions, they further defined the subsets and architecture of immune cells recruited to the sites where the host response contained or did not contain this chronic intracellular infection (35). For example, T cell and myeloid subclusters bearing type I interferon signatures and a myeloid subcluster expressing *TREM2* reflect the altered lipid metabolism and disorganized granulomas that are present primarily in L-lep lesions, which represent immunological failure to clear *M. leprae* (35). In contrast, T cell and myeloid subclusters bearing Th_17_ gene signatures and resembling mature M1 macrophages, respectively, predominate in RR lesions, and these macrophages express genes that contribute to antimicrobial responses (35). In *H. ducreyi*-infected tissue, we identified 12 T- and NK-like cell subtypes (Fig. 3). Pustules contained higher proportions of CD8^+^ subsets (Fig. S3) compared to wounds—including an exhausted T cell subtype and an increased cytotoxic response, which may explain why tissue damage occurs and infection does not resolve in these individuals. We did not observe any Th_17_ cells, which are associated with a resolver phenotype (29), but we did identify Tregs (Fig. 3), which likely dampen the immune response in individuals who form pustules (69).

We identified multiple subtypes of macrophages including macrophage-like cells that formed the ISG-expressing cell cluster. While no macrophage or ISG-expressing cell subcluster had a canonical M1 or M2 phenotype, macrophages found in pustules tended to be more M1-like, and macrophages found in wounds tended to be more M2-like (Fig. 4D). Like Modlin and colleagues, we also identified TREM2^+^ macrophages. While TREM2^+^ macrophages were associated with immunological failure in leprosy (35), most TREM2^+^ macrophages were from wounded sites in our study (Fig. 4C). This suggests that while TREM2^+^ macrophages are associated with failure to contain *M. leprae* infection, they do not appear to have a role in *H. ducreyi* pathogenesis but may have a role in wound healing. As TREM2^+^ macrophages are associated with altered lipid metabolism in several diseases, these cells may be responsible for the alterations in lipid metabolism that we observed by bulk metabolomics in wounds (20). The macrophage-like ISG-expressing cells were located closer to the skin surface above the other macrophages in the pustule where we would expect to find *H. ducreyi*. ISG-expressing cells also had elevated expression of type I interferon (i.e., IFN-⍺/β)- stimulated genes. The one notable exception was of the IDO^+^ TNF^+^ ISG cluster, which was likely responding to type II interferon since these cells expressed *IFNGR2* (Fig. 5). As only CD8^+^ CTLs expressed *IFNG* in our study (Fig. 3), these cell subsets may interact during *H. ducreyi* infection.

*H. ducreyi* in pustules altered the gene expression of stromal cells in the infection microenvironment. Expression of *CXCL14*, *ISG15*, *MX1*, and *WARS* suggests that keratinocyte II cells are in regions where IFN-γ is present. We also identified a small population of myofibroblasts, which are responsible for secreting new collagen. Approximately 80% of myofibroblasts are found in pustules (Fig. S6C), which is consistent with increased levels of TGF-β transcripts and collagen deposition at infected sites (14, 29).

In this study, we compared *H. ducreyi*-infected skin to wounded skin, which differs from the work of Modlin and co-workers where two disease states from chronically infected patients—T-Lep and L-Lep—were compared. In the human challenge model, approximately 26% of volunteers spontaneously clear *H. ducreyi*, while the remaining 74% fail to clear the infection and form pustules (12, 73; Spinola, unpublished). Resolvers and pustule formers who were re-challenged have distinct transcriptomes in tissue collected 48 hours after reinfection and in monocyte-derived dendritic cells that are co-cultured with *H. ducreyi* (29). Using the scRNA-seq and spatial transcriptomics methods and analyses developed in this study, we plan to examine whether dendritic cells or other cell (sub)types drive the transcriptomic differences between reinfected resolvers and pustule formers and whether those transcriptomic differences elucidate the basis for differential host susceptibility; such studies are underway.

## MATERIALS AND METHODS

### Bacterial strain and culture conditions

The *H. ducreyi* strain used in this study was 35000HP, a human passaged isolate of strain 35000 (74). *H. ducreyi* was grown on chocolate agar plates supplemented with 1% IsoVitaleX in the presence of 5% CO_2_. For the human challenge experiments, *H. ducreyi* was grown in a proteose peptone broth-based medium with 1% IsoVitaleX, 5% heat-inactivated fetal calf serum, and 50 µg/mL hemin to mid-log phase. All cultures were grown at 33°C.

### Human volunteers and collection of tissue specimens

Methods for preparation and inoculation of the bacteria, determination of the EDD, clinical observations, biopsies, and antibiotic treatment of the volunteers were performed exactly as described previously (12). Each volunteer was inoculated at three sites on the upper arm with live 35000HP and at one site with a phosphate buffered saline (PBS) control via puncture wounds made by an allergy testing device; the sites were vertically spaced 3 cm apart. Clinical endpoints included the development of a painful pustule, resolution of infection at all sites, or 14 days of observation (12).

Six millimeter punch biopsies were taken of the wound and one to two pustules from five volunteers 7–8 days post inoculation (Table S1). Tissues used for scRNA-seq were transported to the laboratory in Roswell Park Memorial Institute medium (RPMI) + 5% normal human AB serum (NHS) at room temperature and were processed approximately 30 minutes post-collection. Tissue used for Visium spatial transcriptomics were placed in cryomolds, flooded with optimal cutting temperature medium (OCT), and frozen in a liquid nitrogen-cooled isopentane bath at the bedside. Samples were stored on dry ice to allow for residual isopentane evaporation and transported back to the laboratory. Samples were stored at −80°C.

### Single cell suspension generation

Biopsies were washed three times in 10 mL RPMI + 5% NHS. The biopsy was then placed in a petri dish with 1 mL of RPMI and minced with two disposable #10 scalpels into ~1 mm^3^ pieces. The tissue suspension was then added to 9 mL of RPMI containing 25 mg of Collagenase A (Roche) and 3 mg of DNase I 375KU (Sigma) in a 50 mL conical tube. To release the cells, tissue was dissociated at 37°C at 200 rpm in an air shaker for 90 minutes. Cells were pelleted for 5 minutes at 500 × *g*, and the media was removed. One milliliter of TrypLE (Gibco) was added to the pellet, and the tube was shaken at 37°C for an additional 10 minutes. Nine milliliters of PBS + 0.1% bovine serum albumin (BSA) were added to the suspension, which was then passed over a 70 µm cell strainer (Falcon) to remove undigested tissue. The filtrate was centrifuged at 500 × *g* for 5 minutes. The cell pellet was washed twice by suspending it in 5 mL of PBS + 0.1% BSA and pelleting it at 500 × *g* for 5 minutes. The supernatant was removed, and red blood cells were lysed in 2 mL of Gibco ACK Lysing buffer for 10 minutes. Cell debris was removed by two additional washes with 10 mL of PBS + 0.1% BSA. The final cell pellet was suspended in 75 µL of PBS + 0.1% BSA and immediately transported on ice to the Center for Medical Genomics at Indiana University for scRNA-seq library preparation.

### Single cell RNA-sequencing

Cell viability, quality, and concentration was assessed with trypan blue staining. To prevent RNase activity in the cells, 1.5 µL of Protector RNA Inhibitor (Roche) was added to the cells. Cells were then processed using the 10x Genomics Single-cell 3′ RNA-seq Assay. For seven of the samples, approximately 17,000 cells from each cell suspension were loaded on a multiple-channel micro-fluidics chip of the Chromium Single Cell Instrument (10x Genomics) to target 10,000 cells. For three of the samples with lower cell numbers, 5,000–7,000 cells were targeted. Single-cell gel beads in emulsion containing barcoded oligonucleotides and reverse transcriptase reagents were generated with the Next GEM single cell reagent kit (10x Genomics). Following cell capture and cell lysis, cDNA was synthesized and amplified. At each step, the quality of cDNA and library was examined by Bioanalyzer. The final libraries were sequenced on an Illumina NovaSeq 6000. In addition, 100 bp reads including cell barcode and unique molecular identifier (UMI) sequences and 100 bp RNA reads were generated.

### Single cell analysis

CellRanger 5.0.1 (http://support.10xgenomics.com/) was implemented to demultiplex raw base sequence files into sample-specific FASTQ files, which were then aligned to the human reference genome hg19 using RNA-seq aligner STAR. Aligned reads were traced to individual cells and gene expression levels were quantified based on the number of UMIs detected. The filtered barcode matrices were used for further analysis. Cells were retained if they met the following criteria: 100–5,000 unique features/gene counts, <30,000 total reads, and <20% mitochondrial gene reads. Doublets were removed with scDblFinder (75, 76). The total number of cells in each tissue sample before and after filtering is listed in Table S3. Prior to cell clustering analysis, we integrated all remaining cells from all samples using the “RunHarmony” function from the R package “harmony” (77). Based on the integrated data and its top 20 harmony dimensions, we performed clustering using the “FindClusters” function, and UMAP-based 2-D embedding and visualization using “RunUMAP” function from the Seurat package (21). Cluster-specific markers were identified by performing differential gene expression analysis; a Wilcoxon test with a log fold change threshold of 0.25 and false discovery rate <0.05 were used to define significance. The cluster markers were then overlapped to canonical cell type-defining signature genes for cell type annotation. Ultimately, we recovered and annotated 13 unique cell types from the 10 samples. Significant differences between pustules and wounds in each cell (sub)type was determined by Wilcoxon rank-sum test with Benjamini-Hochberg correction for multiple comparisons.

Pathway enrichment analysis was performed by hypergeometric test with pathways collected from the Molecular Signatures Database version 6, including pathways in gene ontology biological process, Reactome, BioCarta, KEGG, and PID (78). Pathways with a false discovery rate-corrected *P*-value of less than 0.05 were considered significantly enriched.

### Cell-cell interaction analysis on scRNA-seq data

Cell-cell interaction analysis was conducted using CellPhoneDB (57) and focused on 12 main cell types, excluding the unknown cluster. Prior to the interaction analysis, transcripts per million (TPM) normalization was applied to the data set. The analysis parameters in CellPhoneDB were set as follows: 1,000 shuffles, a minimum of 10% of cells expressing a gene, five computational threads, a random seed of 42, and a *P*-value threshold of 0.05. Subsampling was enabled, with the subsample size set at one-third of the data set. Upon completion of the cell-cell interaction analysis, *P*-values were adjusted using the Bonferroni method. Self-interactions were filtered out, and circos plots were generated for *TGFB1* (with *TGFBR1*, *TGFBR2*, and *TGFBR3*), *IL1B* (with *IL1R*), and *TNF* (with *TNFRSF1A* and *TNFRSF1B*).

### Spatial transcriptomics sequencing

Spatial transcriptomics were performed using the fresh-frozen protocol for 10x Genomics Visium. The optimal permeabilization time for 10 µm sections of fresh frozen human skin was determined to be 24 minutes. We mounted 10 µm tissue sections on the capture areas of Visium spatial gene expression slides (10x Genomics, Inc). Methanol fixation, H&E staining, and imaging of tissue were carried out according to the 10x Genomics protocol. Visium spatial 3′ gene expression libraries were prepared following the Visium Spatial Gene Expression Reagent Kits User Guide. Briefly, tissue sections fixed on the capture areas of a Visium spatial gene expression slide were permeabilized for 24 minutes, and the cellular mRNAs were captured by the primers on the expression spots. Spatially barcoded full-length cDNAs were synthesized and amplified from polyadenylated mRNA captured on the slide. Illumina sequencing libraries were next prepared from the amplified cDNA through enzymatic fragmentation, size selection, end repair, A-tailing, adaptor ligation, and PCR. Each dual-indexed library was quantified and quality assessed with Qubit and Agilent TapeStation. The final libraries were sequenced on an Illumina NovaSeq 6000.

### Spatial transcriptomics data analysis

Raw sequencing data of spatial transcriptomics were quality checked and mapped by SpaceRanger v.1.1. To maintain the spatial integrity and continuity of the data, no spots were removed. Normalization across spots was performed with the log normalization function in Seurat (21). The total number of spots and average number of genes per spot for each sample is detailed in Table S3.

### Spatial transcriptomics data deconvolution

Since each spatial spot typically contains more than one cell, we applied spatial transcriptomics data deconvolution to disentangle the gene expression signals originating from multiple cell types within each spot. The SPOTlight method is a robust computational technique, leveraging the inherent non-negativity of gene expression profiles to deconvolve mixed signals and estimate cell-type-specific expression patterns, and has been a popular tool used for deconvolution tasks (64). We built our matched cell-type-specific gene expression reference using our single cell RNA-seq data sets for each annotated cell type. Only the top 50 differentially expressed genes for each cell type were used to build the reference expression, and we did so to increase the robustness and specificity of the cell type. Specifically, we first prepared the reference matrix by extracting the mean expression levels of the identified marker genes for each cell type from the single-cell data set. This matrix served as the basis for the subsequent deconvolution. We then used NNLS similar to SPOTlight to deconvolute the spatial transcriptomics data. This involved matching the expression profiles from the spatial data to the reference matrix to determine the proportions of each cell type within each spatial spot. The resulting cell type proportions were then used to create a deconvoluted data set that represented the distribution of various cell types within the tissue samples.

To further refine the deconvoluted data, we performed spatial smoothing of the cell type proportions across the tissue sections with a Gaussian smoothing kernel to ensure a continuous and accurate representation of cell type distributions within the tissue microenvironment. The smoothed data provided a more detailed and precise view of the cellular organization within pustules and wounds.

### Bivariate choropleth mapping to visualizing cell colocalization

To explore the spatial interactions between TNK cells and antigen-presenting cells within patient tissue samples, we utilized a bivariate choropleth mapping approach. This method was applied to visualize the co-localization of different immune cell types in the tissue microenvironment. Here, the first variable represented the proportion of TNK cells, while the second variable corresponded to the proportions of each of the four other antigen-presenting immune cell types: macrophages, mDCs, pDCs, and ISG-expressing cells.

The choropleth map allowed us to easily identify spatial patterns of immune cell distribution and interactions. Regions with strong yellow coloration indicated TNK cell dominance, while those with blue indicated dominance of macrophages, mDC, pDC, or ISG-expressing cells. Areas where TNK cells and the other immune cells co-resided were highlighted by dark green, which resulted from the overlap of high intensities of yellow and blue. These dark green regions provided a clear indication of spatial areas with a high degree of co-localization between TNK cells and antigen-presenting cells. For each cell type, three distinct color intensities represented varying levels of cell type proportions. To achieve this, the proportions from each volunteer’s matching pustule and wound tissues were pooled into a single numeric vector. Fisher’s natural breaks classification was then applied to divide this pooled vector into classes, identifying natural groupings within the data. This method minimizes the variance within each class while maximizing variance between classes, using Fisher’s optimal partitioning algorithm to determine the most distinct boundaries. In this case, we specified three classes for each cell type to capture these natural groupings.

### Local Moran’s I to detect spatially dependent association between TNK cells and APC gene signatures

To further quantify the interaction between T- and NK-like cells and an antigen-presenting cell gene signature intensities, we first estimated the relative levels of antigen presentation at each spatial location in the tissue. This was achieved by calculating the average expression of 88 genes associated with the KEGG antigen processing and presentation pathway. These genes were selected based on their involvement in key steps of antigen processing and presentation, providing a comprehensive measure of antigen presenting cell (APC) activity.

For each spatial tissue sample, we evaluated the spatial correlation between TNK cell proportions and the estimated APC levels using local Moran’s I (79). For each spot, we calculated the local Moran’s I to detect spatial autocorrelation between the estimated TNK cell proportions and antigen presentation levels. The local Moran’s I detects the local spatial autocorrelation between two vectors, i.e., the cell proportion and the estimated APC level vectors.

We first employed a Gaussian smoothing filter to amplify the spatial signals to reduce the discontinuities inherently caused by the low signal level. Specifically, the following smoothing model was used to amplify spatial-dependent signals:


$$f_{i}^{amp}=\sum_{j\in N\left(i\right)}g\left(d\left(i,j\right)\right)*f_{i}$$

where $g\left(x\right)=\frac{1}{\sigma \surd 2\pi }e^{-\frac{{\left(x-\mu \right)}^{2}}{2\sigma^{2}}}$ is a Gaussian kernel and $f_{i}$ represents the deconvoluted cell proportion or estimated APC levels at spatial spot i. N(i) is the neighborhood of i defined as the spatial spots whose distance to i are smaller than a certain threshold, and d(i,j) is the Euclidean distance between two spatial spots i and j, and $f_{i}^{amp}$ is the amplified signal of spot i. Here, we utilized all the spatial spots to define the neighborhood of each spot. Then, we computed local bivariate Moran’s I correlation (79) for each spatial spot i:


$$I_{i}=\frac{\sum_{j}w_{ij}y_{j}\times x_{i}}{\sum_{i}x_{i}^{2}}$$

where $x_{i}$ represents the estimated APC level after amplification at spot i, $y_{j}$ represents the deconvoluted TNK cell proportions after amplification at spot j, and $w_{ij}$ is a weight indexing location of spot i relative to j. In this study, we used the Gaussian kernel distance to compute $w_{ij}$.

For each spot i, $I_{i}$ measures the correlation between its APC level ($x_{i}$) with the level of TNK cell abundance ($y_{i}$) in its neighborhood [$\sum_{j\in N(i)}w_{ij}y_{j}$]. The significance of $I_{i}$ was evaluated using a permutation test, where the $y_{j}$ values were randomly permuted across all spots. A pseudo *P*-value was then calculated by determining the proportion of local Moran’s I values generated from permutations that are greater than or equal to the observed local Moran’s I values from the original data. We set a significance level of α = 0.05, and the spots with a pseudo *P*-value *P* < 0.05 were considered statistically significant, indicating significant local spatial dependence between APC levels and TNK cell infiltration level.

Next, we segmented the spatial regions into five distinct regions: (i) high TNK and high APC levels, (ii) high TNK and low APC levels, (iii) low TNK and high APC levels, (iv) low TNK and low APC levels, and (v) regions without significant spatial autocorrelation. These segmentations were based on the APC level of each spot [z-score normalized $x_{i}$, denoted as ${z(x}_{i})$] and the average TNK cell level of its neighbors [weighted average z-score of $y_{j}$, denoted as ${w_{ij}z(y}_{j})$], where z-score is computed by standardizing the variable of interest (subtracting the mean and dividing by the standard deviation). Again, the z-scores from the same volunteer’s matching pustule and wound sites were pooled, and the pooled vector was divided into two classes—high and low—using Fisher’s natural breaks classification. For spatial spots with significant spatial autocorrelation (pseudo *P*-value <0.05), “High TNK-High APC” regions were identified as spots classified as having high-antigen presentation levels, with neighboring regions showing TNK cell infiltration also classified as high. The other regions—“High TNK-Low APC,” “Low TNK-High APC,” and “Low TNK-Low APC”—follow a similar interpretation. The spatial spots with pseudo *P*-value larger than 0.05 were classified as non-significant.

### Local Moran’s I to detect spatially dependent cell-cell interactions

To detect cell-cell interactions on the spatial domain, we utilized the same local Moran’s I method and Fisher’s natural breaks classification as outlined above. Note that the high and low classes were always performed after pooling spots from the pustule and matching wound sample from the same volunteer, similar to the method above. Here, similarly, for each pair of the 13 cell types and for each spatial sample, the spatial regions was dissected into five regions: (i) high cell type 1 and high cell type 2, (ii) high cell type 1 and low cell type 2, (iii) low cell type 1 and high cell type 2, (iv) low cell type 1 and low cell type 2, and (v) regions without significant spatial autocorrelation. We further combined classes ii and iii, termed as negative interaction, and classes iv and v, termed as no interaction. We call class i a positive interaction region. The proportions of the class I patterns were calculated, and the resulting average proportions across pustule and wound samples and are shown in Table S2. The *P*-value for testing the difference of proportions was determined by *t*-test.

## Data Availability

The raw RNA-seq count tables and sequence read files related to this study have been deposited in NCBI dbGaP under accession number phs003754.

## SUPPLEMENTAL MATERIAL

Data Set S1 mbio.03885-24-s0001.xlsx Top 30 DEGs for select cell subsets.

Data Set S2 mbio.03885-24-s0002.xlsx Differential gene expression in pustules versus wounds in areas of high antigen presentation.

Supplemental Figures mbio.03885-24-s0003.pdf Figures S1-S11.

Table S1 mbio.03885-24-s0004.docx Response to inoculation with *H. ducreyi* 35000HP.

Table S2 mbio.03885-24-s0005.xlsx Proportion of overlapping cell-cell interactions.

Table S3 mbio.03885-24-s0006.docx Number of single cells before and after filtering and number of spatial spots and average number of genes per spot.

ASM does not own the copyrights to Supplemental Material that may be linked to, or accessed through, an article. The authors have granted ASM a non-exclusive, world-wide license to publish the Supplemental Material files. Please contact the corresponding author directly for reuse.

## References

[1] González-Beiras C, Marks M, Chen CY, Roberts S, Mitjà O. 2016. Epidemiology of Haemophilus ducreyi infections. Emerg Infect Dis 22:1–8. doi:10.3201/eid2201.150425 26694983 PMC4696685

[2] Tshaka TR, Singh R, Apalata TR, Mbulawa ZZA. 2022. Aetiology of genital ulcer disease and associated factors among Mthatha public clinic attendees. S Afr J Infect Dis 37:444. doi:10.4102/sajid.v37i1.444 36568332 PMC9772736

[3] Chen JS, Matoga MM, Gaither CF, Jere E, Mathiya E, Bonongwe N, Krysiak R, Banda G, Hoffman IF, Miller WC, Juliano JJ, Rutstein SE. 2023. Dramatic shift in the etiology of genital ulcer disease among patients visiting a sexually transmitted infections clinic in Lilongwe, Malawi. Sex Transm Dis 50:753–759. doi:10.1097/OLQ.0000000000001853 37824787 PMC10575672

[4] Al-Tawfiq JA, Spinola SM. 2024. Infections caused by Haemophilus ducreyi: one organism, two stories. Clin Microbiol Rev 37:e0013524. doi:10.1128/cmr.00135-24 39287406 PMC11629627

[5] Mitjà O, Lukehart SA, Pokowas G, Moses P, Kapa A, Godornes C, Robson J, Cherian S, Houinei W, Kazadi W, Siba P, de Lazzari E, Bassat Q. 2014. Haemophilus ducreyi as a cause of skin ulcers in children from a yaws-endemic area of Papua New Guinea: a prospective cohort study. Lancet Glob Health 2:e235–41. doi:10.1016/S2214-109X(14)70019-1 25103064

[6] Akuffo RA, Sanchez C, Amanor I, Amedior JS, Kotey NK, Anto F, Azurago T, Ablordey A, Owusu-Antwi F, Beshah A, Amoako YA, Phillips RO, Wilson M, Asiedu K, Ruiz-Postigo JA, Moreno J, Mokni M. 2023. Endemic infectious cutaneous ulcers syndrome in the Oti Region of Ghana: Study of cutaneous leishmaniasis, yaws and Haemophilus ducreyi cutaneous ulcers. PLoS One 18:e0292034. doi:10.1371/journal.pone.0292034 37756291 PMC10529585

[7] Ndzomo P, Tchatchouang S, Njih Tabah E, Njamnshi T, Tsanga MVN, Bondi JA, Handley R, González Beiras C, Tchatchueng J, Müller C, Lüert S, Knauf S, Boyomo O, Harding-Esch E, Mitja O, Crucitti T, Marks M, Eyangoh S. 2023. Prevalence and risk factors associated with Haemophilus ducreyi cutaneous ulcers in cameroon. PLoS Negl Trop Dis 17:e0011553. doi:10.1371/journal.pntd.0011553 38150487 PMC10791135

[8] Ndzomo Ngono J-P, Tchatchouang S, Noah Tsanga MV, Njih Tabah E, Tchualeu A, Asiedu K, Giacani L, Eyangoh S, Crucitti T. 2021. Ulcerative skin lesions among children in Cameroon: it is not always yaws. PLoS Negl Trop Dis 15:e0009180. doi:10.1371/journal.pntd.0009180 33591973 PMC7909670

[9] Houinei W, Godornes C, Kapa A, Knauf S, Mooring EQ, González-Beiras C, Watup R, Paru R, Advent P, Bieb S, Sanz S, Bassat Q, Spinola SM, Lukehart SA, Mitjà O. 2017. Haemophilus ducreyi DNA is detectable on the skin of asymptomatic children, flies and fomites in villages of Papua New Guinea. PLoS Negl Trop Dis 11:e0004958. doi:10.1371/journal.pntd.0004958 28489855 PMC5425006

[10] Deli J, González-Beiras C, Guldan GS, Moses RL, Dally J, Moseley R, Lundy FT, Corbacho-Monne M, Walker SL, Cazorla MU, Ouchi D, Fang R, Briggs M, Kiapranis R, Yahimbu M, Mitjà O, Prescott TAK. 2022. Ficus septica exudate, a traditional medicine used in Papua New Guinea for treating infected cutaneous ulcers: in vitro evaluation and clinical efficacy assessment by cluster randomised trial. Phytomedicine 99:154026. doi:10.1016/j.phymed.2022.154026 35278903

[11] Spinola SM, Bauer ME, Munson RS Jr. 2002. Immunopathogenesis of Haemophilus ducreyi infection (chancroid). Infect Immun 70:1667–1676. doi:10.1128/IAI.70.4.1667-1676.2002 11895928 PMC127820

[12] Janowicz DM, Ofner S, Katz BP, Spinola SM. 2009. Experimental infection of human volunteers with Haemophilus ducreyi: fifteen years of clinical data and experience. J Infect Dis 199:1671–1679. doi:10.1086/598966 19432549 PMC2682218

[13] Brothwell JA, Griesenauer B, Chen L, Spinola SM. 2020. Interactions of the skin pathogen Haemophilus ducreyi with the human host. Front Immunol 11:615402. doi:10.3389/fimmu.2020.615402 33613541 PMC7886810

[14] Bauer ME, Goheen MP, Townsend CA, Spinola SM. 2001. Haemophilus ducreyi associates with phagocytes, collagen, and fibrin and remains extracellular throughout infection of human volunteers. Infect Immun 69:2549–2557. doi:10.1128/IAI.69.4.2549-2557.2001 11254619 PMC98191

[15] Palmer KL, Schnizlein-Bick CT, Orazi A, John K, Chen C-Y, Hood AF, Spinola SM. 1998. The immune response to Haemophilus ducreyi resembles a delayed-type hypersensitivity reaction throughout experimental infection of human subjects. J Infect Dis 178:1688–1697. doi:10.1086/314489 9815221

[16] Banks KE, Humphreys TL, Li W, Katz BP, Wilkes DS, Spinola SM. 2007. Haemophilus ducreyi partially activates human myeloid dendritic cells. Infect Immun 75:5678–5685. doi:10.1128/IAI.00702-07 17923525 PMC2168323

[17] Humphreys TL, Baldridge LA, Billings SD, Campbell JJ, Spinola SM. 2005. Trafficking pathways and characterization of CD4 and CD8 cells recruited to the skin of humans experimentally infected with Haemophilus ducreyi*.* Infect Immun 73:3896–3902. doi:10.1128/IAI.73.7.3896-3902.2005 15972475 PMC1168611

[18] Li W, Janowicz DM, Fortney KR, Katz BP, Spinola SM. 2009. Mechanism of human natural killer cell activation by Haemophilus ducreyi. J Infect Dis 200:590–598. doi:10.1086/600123 19572804 PMC2768539

[19] Bauer ME, Townsend CA, Ronald AR, Spinola SM. 2006. Localization of Haemophilus ducreyi in naturally acquired chancroidal ulcers. Microbes Infect 8:2465–2468. doi:10.1016/j.micinf.2006.06.001 16872858

[20] Brothwell JA, Fortney KR, Gao H, Wilson LS, Andrews CF, Tran TM, Hu X, Batteiger TA, Barnes S, Liu Y, Spinola SM. 2022. Haemophilus ducreyi infection induces oxidative stress, central metabolic changes, and a mixed pro- and anti-inflammatory environment in the human host. MBio 13:e0312522. doi:10.1128/mbio.03125-22 36453940 PMC9765465

[21] Hao Y, Stuart T, Kowalski MH, Choudhary S, Hoffman P, Hartman A, Srivastava A, Molla G, Madad S, Fernandez-Granda C, Satija R. 2024. Dictionary learning for integrative, multimodal and scalable single-cell analysis. Nat Biotechnol 42:293–304. doi:10.1038/s41587-023-01767-y 37231261 PMC10928517

[22] Ianevski A, Giri AK, Aittokallio T. 2022. Fully-automated and ultra-fast cell-type identification using specific marker combinations from single-cell transcriptomic data. Nat Commun 13:1246. doi:10.1038/s41467-022-28803-w 35273156 PMC8913782

[23] Humphreys TL, Schnizlein-Bick CT, Katz BP, Baldridge LA, Hood AF, Hromas RA, Spinola SM. 2002. Evolution of the cutaneous immune response to experimental Haemophilus ducreyi infection and its relevance to HIV-1 acquisition. J Immunol 169:6316–6323. doi:10.4049/jimmunol.169.11.6316 12444138

[24] Wang W, Lopez McDonald MC, Kim C, Ma M, Pan ZT, Kaufmann C, Frank DA. 2023. The complementary roles of STAT3 and STAT1 in cancer biology: insights into tumor pathogenesis and therapeutic strategies. Front Immunol 14:1265818. doi:10.3389/fimmu.2023.1265818 38022653 PMC10663227

[25] Zenke K, Muroi M, Tanamoto KI. 2018. IRF1 supports DNA binding of STAT1 by promoting its phosphorylation. Immunol Cell Biol 96:1095–1103. doi:10.1111/imcb.12185 29893425

[26] Zhang H, Bernaleau L, Delacrétaz M, Hasanovic E, Drobek A, Eibel H, Rebsamen M. 2023. SLC15A4 controls endolysosomal TLR7–9 responses by recruiting the innate immune adaptor TASL. Cell Rep 42:112916. doi:10.1016/j.celrep.2023.112916 37527038

[27] Chen X, Xie M, Zhang S, Monguió-Tortajada M, Yin J, Liu C, Zhang Y, Delacrétaz M, Song M, Wang Y, Dong L, Ding Q, Zhou B, Tian X, Deng H, Xu L, Liu X, Yang Z, Chang Q, Na J, Zeng W, Superti-Furga G, Rebsamen M, Yang M. 2023. Structural basis for recruitment of TASL by SLC15A4 in human endolysosomal TLR signaling. Nat Commun 14:6627. doi:10.1038/s41467-023-42210-9 37863913 PMC10589346

[28] Singer M, Li W, Morré SA, Ouburg S, Spinola SM. 2016. Host polymorphisms in TLR9 and IL10 are associated with the outcomes of experimental Haemophilus ducreyi infection in human volunteers. J Infect Dis 214:489–495. doi:10.1093/infdis/jiw164 27122592 PMC4936646

[29] Humphreys TL, Li L, Li X, Janowicz DM, Fortney KR, Zhao Q, Li W, McClintick J, Katz BP, Wilkes DS, Edenberg HJ, Spinola SM. 2007. Dysregulated immune profiles for skin and dendritic cells are associated with increased host susceptibility to Haemophilus ducreyi infection in human volunteers. Infect Immun 75:5686–5697. doi:10.1128/IAI.00777-07 17893130 PMC2168359

[30] Spinola SM, Orazi A, Arno JN, Fortney K, Kotylo P, Chen C-Y, Campagnari AA, Hood AF. 1996. Haemophilus ducreyi elicits a cutaneous infiltrate of CD4 cells during experimental human infection. J Infect Dis 173:394–402. doi:10.1093/infdis/173.2.394 8568301

[31] Clark RA, Kupper TS. 2007. IL-15 and dermal fibroblasts induce proliferation of natural regulatory T cells isolated from human skin. Blood 109:194–202. doi:10.1182/blood-2006-02-002873 16968902 PMC1785078

[32] Sallusto F, Lenig D, Förster R, Lipp M, Lanzavecchia A. 1999. Two subsets of memory T lymphocytes with distinct homing potentials and effector functions. Nature 401:708–712. doi:10.1038/44385 10537110

[33] Gelfanova V, Humphreys TL, Spinola SM. 2001. Characterization of Haemophilus ducreyi-specific T-cell lines from lesions of experimentally infected human subjects. Infect Immun 69:4224–4231. doi:10.1128/IAI.69.7.4224-4231.2001 11401958 PMC98455

[34] Li W, Katz BP, Spinola SM. 2012. Haemophilus ducreyi-induced interleukin-10 promotes a mixed M1 and M2 activation program in human macrophages. Infect Immun 80:4426–4434. doi:10.1128/IAI.00912-12 23027536 PMC3497422

[35] Ma F, Hughes TK, Teles RMB, Andrade PR, de Andrade Silva BJ, Plazyo O, Tsoi LC, Do T, Wadsworth MH II, Oulee A, Ochoa MT, Sarno EN, Iruela-Arispe ML, Klechevsky E, Bryson B, Shalek AK, Bloom BR, Gudjonsson JE, Pellegrini M, Modlin RL. 2021. The cellular architecture of the antimicrobial response network in human leprosy granulomas. Nat Immunol 22:839–850. doi:10.1038/s41590-021-00956-8 34168371 PMC8579511

[36] Nimmerjahn F, Ravetch JV. 2008. Fcgamma receptors as regulators of immune responses. Nat Rev Immunol 8:34–47. doi:10.1038/nri2206 18064051

[37] Chen CY, Mertz KJ, Spinola SM, Morse SA. 1997. Comparison of enzyme immunoassays for antibodies to Haemophilus ducreyi in a community outbreak of chancroid in the United States. J Infect Dis 175:1390–1395. doi:10.1086/516471 9180178

[38] Dodd DA, Worth RG, Rosen MK, Grinstein S, van Oers NSC, Hansen EJ. 2014. The Haemophilus ducreyi LspA1 protein inhibits phagocytosis by using a new mechanism involving activation of C-terminal Src kinase. MBio 5:e01178-14. doi:10.1128/mBio.01178-14 24902122 PMC4030455

[39] Li W, Katz BP, Spinola SM. 2011. Haemophilus ducreyi lipooligosaccharides induce expression of the immunosuppressive enzyme indoleamine 2,3-dioxygenase via type I interferons and tumor necrosis factor alpha in human dendritic cells. Infect Immun 79:3338–3347. doi:10.1128/IAI.05021-11 21576329 PMC3147552

[40] Puddu P, Carollo M, Pietraforte I, Spadaro F, Tombesi M, Ramoni C, Belardelli F, Gessani S. 2005. IL-2 induces expression and secretion of IFN-gamma in murine peritoneal macrophages. J Leukoc Biol 78:686–695. doi:10.1189/jlb.0105035 15951352

[41] Bosco MC, Curiel RE, Zea AH, Malabarba MG, Ortaldo JR, Espinoza-Delgado I. 2000. IL-2 signaling in human monocytes involves the phosphorylation and activation of p59^hck^. J Immunol 164:4575–4585. doi:10.4049/jimmunol.164.9.4575 10779760

[42] Suwanpradid J, Lee MJ, Hoang P, Kwock J, Floyd LP, Smith JS, Yin Z, Atwater AR, Rajagopal S, Kedl RM, Corcoran DL, Zhang JY, MacLeod AS. 2021. IL-27 derived from macrophages facilitates IL-15 production and T cell maintenance following allergic hypersensitivity responses. Front Immunol 12:713304. doi:10.3389/fimmu.2021.713304 34659203 PMC8515907

[43] Chang M. 2023. Matrix metalloproteinase profiling and their roles in disease. RSC Adv 13:6304–6316. doi:10.1039/d2ra07005g 36825288 PMC9942564

[44] Nguyen TT, Ding D, Wolter WR, Pérez RL, Champion MM, Mahasenan KV, Hesek D, Lee M, Schroeder VA, Jones JI, Lastochkin E, Rose MK, Peterson CE, Suckow MA, Mobashery S, Chang M. 2018. Validation of matrix metalloproteinase-9 (MMP-9) as a novel target for treatment of diabetic foot ulcers in humans and discovery of a potent and selective small-molecule MMP-9 inhibitor that accelerates healing. J Med Chem 61:8825–8837. doi:10.1021/acs.jmedchem.8b01005 30212201

[45] Dinarello CA. 2019. The IL-1 family of cytokines and receptors in rheumatic diseases. Nat Rev Rheumatol 15:612–632. doi:10.1038/s41584-019-0277-8 31515542

[46] Li W, Katz BP, Bauer ME, Spinola SM. 2013. Haemophilus ducreyi infection induces activation of the NLRP3 inflammasome in nonpolarized but not in polarized human macrophages. Infect Immun 81:2997–3008. doi:10.1128/IAI.00354-13 23753629 PMC3719567

[47] Milacic M, Beavers D, Conley P, Gong C, Gillespie M, Griss J, Haw R, Jassal B, Matthews L, May B, Petryszak R, Ragueneau E, Rothfels K, Sevilla C, Shamovsky V, Stephan R, Tiwari K, Varusai T, Weiser J, Wright A, Wu G, Stein L, Hermjakob H, D’Eustachio P. 2024. The reactome pathway knowledgebase 2024. Nucleic Acids Res 52:D672–D678. doi:10.1093/nar/gkad1025 37941124 PMC10767911

[48] Shah A, Lindquist JA, Rosendahl L, Schmitz I, Mertens PR. 2021. Novel insights into YB-1 signaling and cell death decisions. Cancers (Basel) 13:3306. doi:10.3390/cancers13133306 34282755 PMC8269159

[49] Prabhu L, Hartley A-V, Martin M, Warsame F, Sun E, Lu T. 2015. Role of post-translational modification of the Y box binding protein 1 in human cancers. Genes Dis 2:240–246. doi:10.1016/j.gendis.2015.05.001 30258867 PMC6150071

[50] Zhang X, Yin M, Zhang L. 2019. Keratin 6, 16 and 17—critical barrier alarmin molecules in skin wounds and psoriasis. Cells 8:807. doi:10.3390/cells8080807 31374826 PMC6721482

[51] Elliott CG, Wang J, Guo X, Xu S, Eastwood M, Guan J, Leask A, Conway SJ, Hamilton DW. 2012. Periostin modulates myofibroblast differentiation during full-thickness cutaneous wound repair. J Cell Sci 125:121–132. doi:10.1242/jcs.087841 22266908 PMC3269025

[52] Gerrits T, Zandbergen M, Wolterbeek R, Bruijn JA, Baelde HJ, Scharpfenecker M. 2020. Endoglin promotes myofibroblast differentiation and extracellular matrix production in diabetic nephropathy. Int J Mol Sci 21:7713. doi:10.3390/ijms21207713 33081058 PMC7589772

[53] Wiedemann J, Billi AC, Bocci F, Kashgari G, Xing E, Tsoi LC, Meller L, Swindell WR, Wasikowski R, Xing X, Ma F, Gharaee-Kermani M, Kahlenberg JM, Harms PW, Maverakis E, Nie Q, Gudjonsson JE, Andersen B. 2023. Differential cell composition and split epidermal differentiation in human palm, sole, and hip skin. Cell Rep 42:111994. doi:10.1016/j.celrep.2023.111994 36732947 PMC9939370

[54] Hays RC, Mandell GL. 1974. PO_2_, pH, and redox potential of experimental abscesses. Proc Soc Exp Biol Med 147:29–30. doi:10.3181/00379727-147-38275 4612550

[55] Brothwell JA, Spinola SM. 2022. Genes differentially expressed by Haemophilus ducreyi during anaerobic growth significantly overlap those differentially expressed during experimental infection of human volunteers. J Bacteriol 204:e0000522. doi:10.1128/jb.00005-22 35377183 PMC9112927

[56] Garcia-Alonso L, Lorenzi V, Mazzeo CI, Alves-Lopes JP, Roberts K, Sancho-Serra C, Engelbert J, Marečková M, Gruhn WH, Botting RA, Li T, Crespo B, van Dongen S, Kiselev VY, Prigmore E, Herbert M, Moffett A, Chédotal A, Bayraktar OA, Surani A, Haniffa M, Vento-Tormo R. 2022. Single-cell roadmap of human gonadal development. Nature 607:540–547. doi:10.1038/s41586-022-04918-4 35794482 PMC9300467

[57] Efremova M, Vento-Tormo M, Teichmann SA, Vento-Tormo R. 2020. CellPhoneDB: inferring cell-cell communication from combined expression of multi-subunit ligand-receptor complexes. Nat Protoc 15:1484–1506. doi:10.1038/s41596-020-0292-x 32103204

[58] Annes JP, Chen Y, Munger JS, Rifkin DB. 2004. Integrin alphaVbeta6-mediated activation of latent TGF-beta requires the latent TGF-beta binding protein-1. J Cell Biol 165:723–734. doi:10.1083/jcb.200312172 15184403 PMC2172370

[59] Liarte S, Bernabé-García Á, Nicolás FJ. 2020. Role of TGF-β in skin chronic wounds: a keratinocyte perspective. Cells 9:306. doi:10.3390/cells9020306 32012802 PMC7072438

[60] Häkkinen L, Koivisto L, Gardner H, Saarialho-Kere U, Carroll JM, Lakso M, Rauvala H, Laato M, Heino J, Larjava H. 2004. Increased expression of beta6-integrin in skin leads to spontaneous development of chronic wounds. Am J Pathol 164:229–242. doi:10.1016/s0002-9440(10)63113-6 14695336 PMC1602209

[61] Desmoulière A, Geinoz A, Gabbiani F, Gabbiani G. 1993. Transforming growth factor-beta 1 induces alpha-smooth muscle actin expression in granulation tissue myofibroblasts and in quiescent and growing cultured fibroblasts. J Cell Biol 122:103–111. doi:10.1083/jcb.122.1.103 8314838 PMC2119614

[62] Pakyari M, Farrokhi A, Maharlooei MK, Ghahary A. 2013. Critical role of transforming growth factor beta in different phases of wound healing. Adv Wound Care (New Rochelle) 2:215–224. doi:10.1089/wound.2012.0406 24527344 PMC3857353

[63] Hobbs MM, Paul TR, Wyrick PB, Kawula TH. 1998. Haemophilus ducreyi infection causes basal keratinocyte cytotoxicity and elicits a unique cytokine induction pattern in an in vitro human skin model. Infect Immun 66:2914–2921. doi:10.1128/IAI.66.6.2914-2921.1998 9596767 PMC108289

[64] Elosua-Bayes M, Nieto P, Mereu E, Gut I, Heyn H. 2021. SPOTlight: seeded NMF regression to deconvolute spatial transcriptomics spots with single-cell transcriptomes. Nucleic Acids Res 49:e50. doi:10.1093/nar/gkab043 33544846 PMC8136778

[65] Westermann A.J, Gorski SA, Vogel J. 2012. Dual RNA-seq of pathogen and host. Nat Rev Microbiol 10:618–630. doi:10.1038/nrmicro2852 22890146

[66] Westermann AJ, Förstner KU, Amman F, Barquist L, Chao Y, Schulte LN, Müller L, Reinhardt R, Stadler PF, Vogel J. 2016. Dual RNA-seq unveils noncoding RNA functions in host-pathogen interactions. Nature 529:496–501. doi:10.1038/nature16547 26789254

[67] Westermann A.J, Barquist L, Vogel J. 2017. Resolving host-pathogen interactions by dual RNA-seq. PLoS Pathog 13:e1006033. doi:10.1371/journal.ppat.1006033 28207848 PMC5313147

[68] Soler D, Humphreys TL, Spinola SM, Campbell JJ. 2003. CCR4 versus CCR10 in human cutaneous TH lymphocyte trafficking. Blood 101:1677–1682. doi:10.1182/blood-2002-07-2348 12406880

[69] Li W, Tenner-Racz K, Racz P, Janowicz DM, Fortney KR, Katz BP, Spinola SM. 2010. Role played by CD4^+^FOXP3^+^ regulatory T Cells in suppression of host responses to Haemophilus ducreyi during experimental infection of human volunteers. J Infect Dis 201:1839–1848. doi:10.1086/652781 20443736 PMC2873097

[70] Bauer ME, Spinola SM. 2000. Localization of Haemophilus ducreyi at the pustular stage of disease in the human model of infection. Infect Immun 68:2309–2314. doi:10.1128/IAI.68.4.2309-2314.2000 10722634 PMC97418

[71] Bauer ME, Fortney KR, Harrison A, Janowicz DM, Munson RS, Spinola SM. 2008. Identification of Haemophilus ducreyi genes expressed during human infection. Microbiology (Reading) 154:1152–1160. doi:10.1099/mic.0.2007/013953-0 18375807 PMC2852322

[72] Montoya DJ, Andrade P, Silva BJA, Teles RMB, Ma F, Bryson B, Sadanand S, Noel T, Lu J, Sarno E, Arnvig KB, Young D, Lahiri R, Williams DL, Fortune S, Bloom BR, Pellegrini M, Modlin RL. 2019. Dual RNA-Seq of human leprosy lesions identifies bacterial determinants linked to host immune response. Cell Rep 26:3574–3585. doi:10.1016/j.celrep.2019.02.109 30917313 PMC6508871

[73] Janowicz DM, Li W, Bauer ME. 2010. Host-pathogen interplay of Haemophilus ducreyi. Curr Opin Infect Dis 23:64–69. doi:10.1097/QCO.0b013e328334c0cb 19918177 PMC2867464

[74] Al-Tawfiq JA, Thornton AC, Katz BP, Fortney KR, Todd KD, Hood AF, Spinola SM. 1998. Standardization of the experimental model of Haemophilus ducreyi infection in human subjects. J Infect Dis 178:1684–1687. doi:10.1086/314483 9815220

[75] Xi NM, Li JJ. 2021. Protocol for executing and benchmarking eight computational doublet-detection methods in single-cell RNA sequencing data analysis. STAR Protoc 2:100699. doi:10.1016/j.xpro.2021.100699 34382023 PMC8339294

[76] Germain PL, Lun A, Garcia Meixide C, Macnair W, Robinson MD. 2021. Doublet identification in single-cell sequencing data using scDblFinder. F1000Res 10:979. doi:10.12688/f1000research.73600.2 35814628 PMC9204188

[77] Korsunsky I, Millard N, Fan J, Slowikowski K, Zhang F, Wei K, Baglaenko Y, Brenner M, Loh PR, Raychaudhuri S. 2019. Fast, sensitive and accurate integration of single-cell data with Harmony. Nat Methods 16:1289–1296. doi:10.1038/s41592-019-0619-0 31740819 PMC6884693

[78] Subramanian A, Tamayo P, Mootha VK, Mukherjee S, Ebert BL, Gillette MA, Paulovich A, Pomeroy SL, Golub TR, Lander ES, Mesirov JP. 2005. Gene set enrichment analysis: a knowledge-based approach for interpreting genome-wide expression profiles. Proc Natl Acad Sci U S A 102:15545–15550. doi:10.1073/pnas.0506580102 16199517 PMC1239896

[79] Lee S-I. 2001. Developing a bivariate spatial association measure: an integration of Pearson’s r and Moran’s I. J Geogr Syst 3:369–385. doi:10.1007/s101090100064

