# Supplementary material for: A high-resolution view of the immune and stromal cell response to Haemophilus ducreyi infection in human volunteers
Source: mBio. 2025 Jan 30;16(3):e03885-24. doi: 10.1128/mbio.03885-24 (PMC11898715; doi:10.1128/mbio.03885-24)
Supplement: Supplemental Figures — Figures S1-S11. [file mbio.03885-24-s0003.pdf]

A

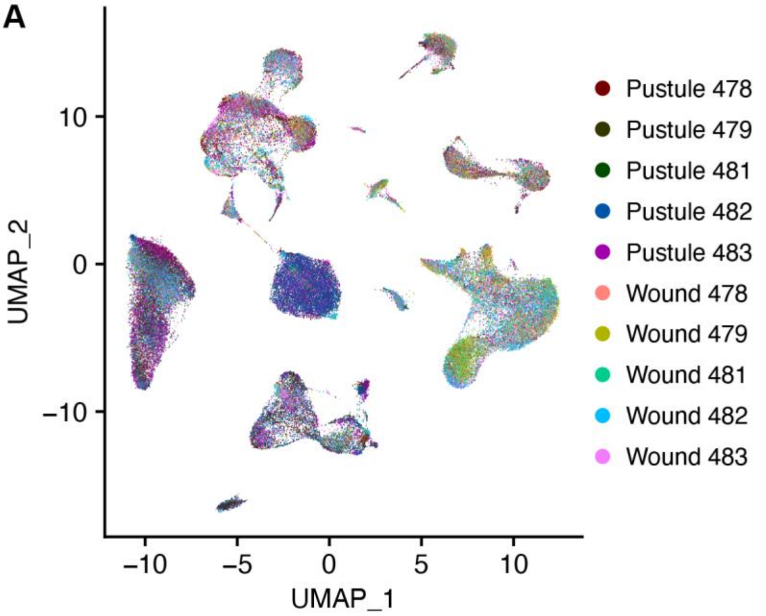

B

Cell Type	Pustule	Wound
T- and NK-like cells	11240	5875
ISG-expressing cells	9943	824
Fibroblasts	2651	16301
Macrophages	3722	1595
Keratinocytes	5614	9497
SMCs	1805	3796
mDCs	1772	1029
Endothelial cells	1047	2120
Melanocytes	180	1033
Mast cells	228	880
pDCs	772	75
B cells	719	16
Unknown	210	28

C

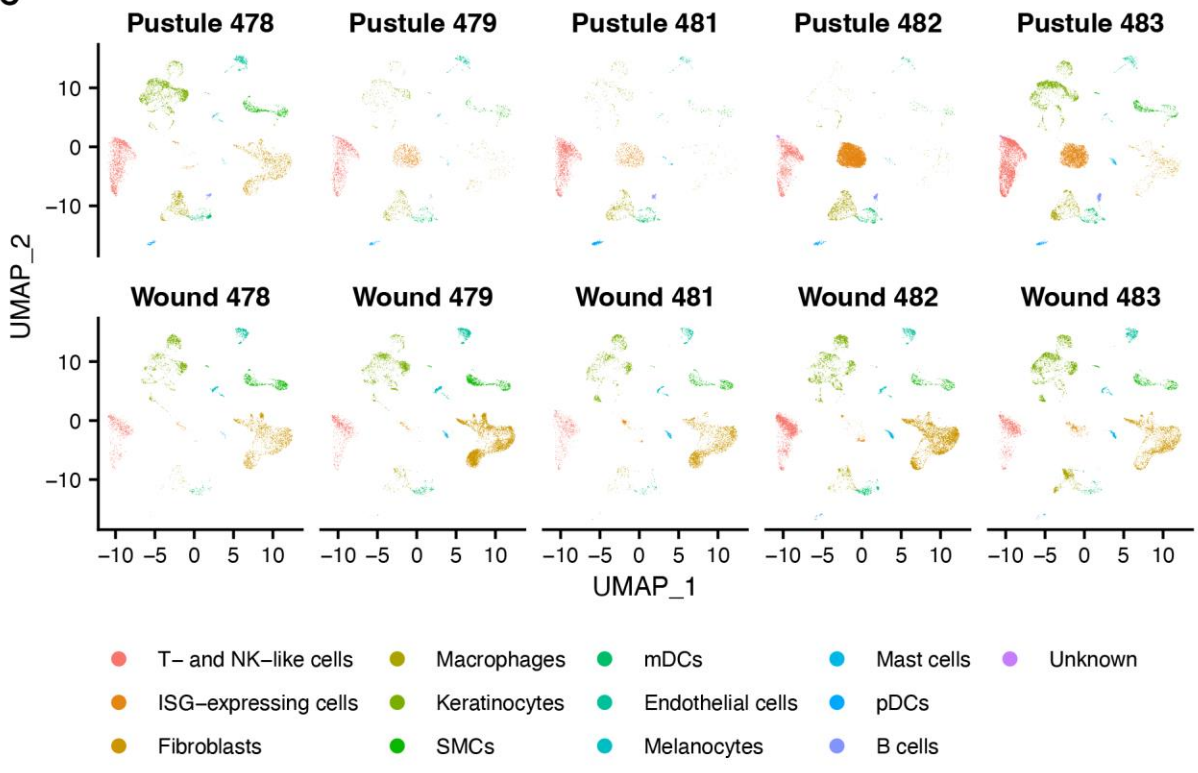

FIG S1 Integration of individual datasets. (**A**) UMAP showing the integration of samples. Each sample is a different color. (**B**) The number of cells contributed to the UMAP by each sample. (**C**) The distribution of cells from each sample in (A) is shown for individual samples. Coloring corresponds to the major cell types identified in FIG 1.

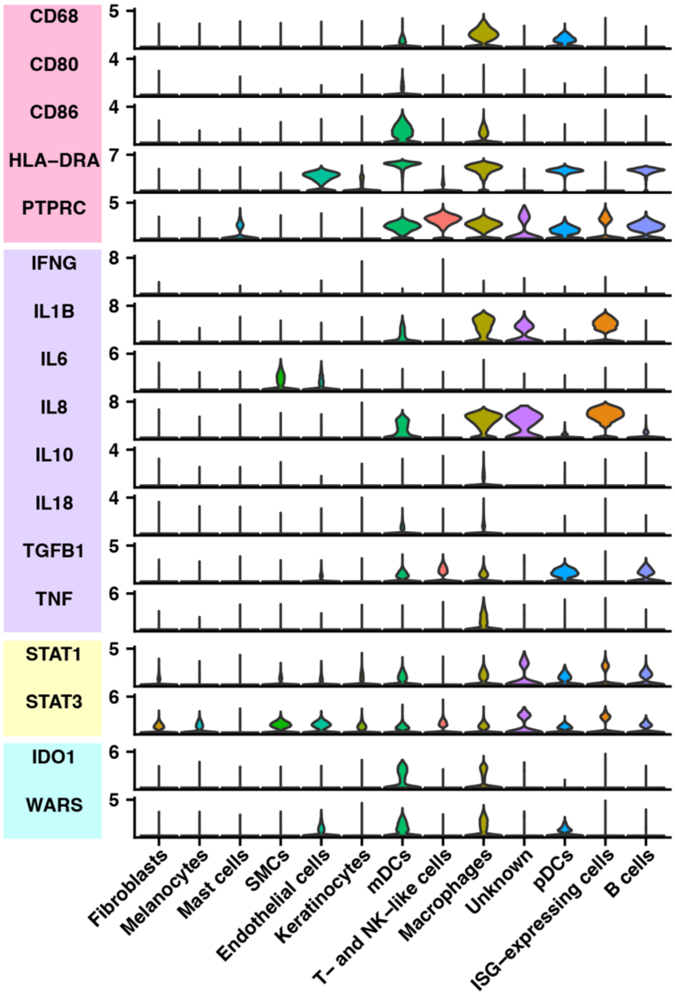

FIG S2 Expression of immune activation genes. Genes that encode markers of immune cells (*PTPRC*/*CD45*) and antigen presentation on antigen presenting cells (pink box) and various cytokines (e.g., IL-6, IL-8, IL-18, and TNF; purple box) and are displayed. Several cytokines have multiple endogenous cell sources. Genes encoding select transcription factors (yellow boxes) and interferon-stimulated genes (blue box) are also displayed.

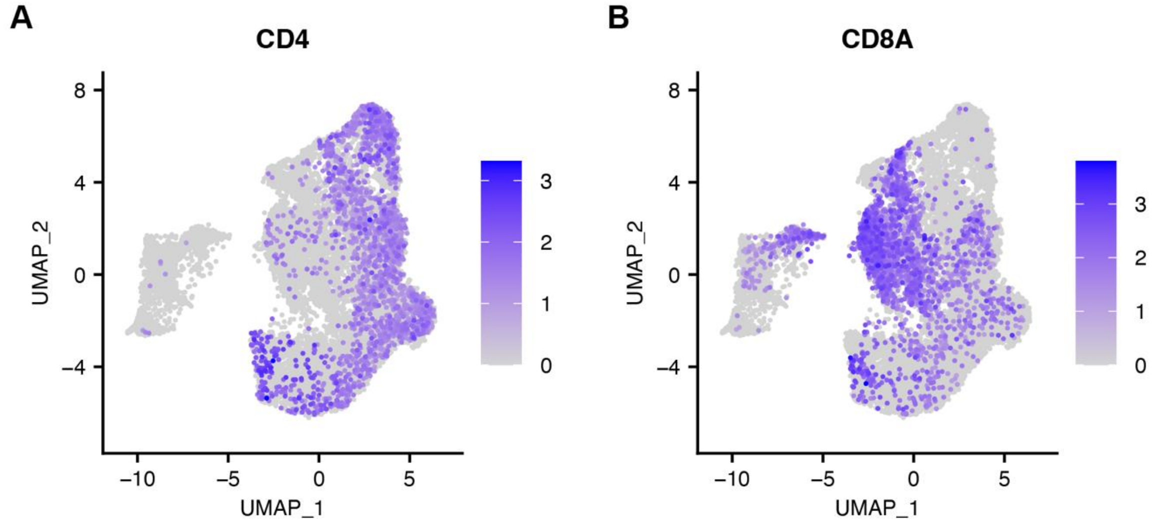

FIG S3 *CD4* and *CD8* expression in T- and NK-like cells. UMAP plots of (**A**) *CD4* and (**B**) *CD8A* expression in the T- and NK-like cell clusters identified in Figure 3A.

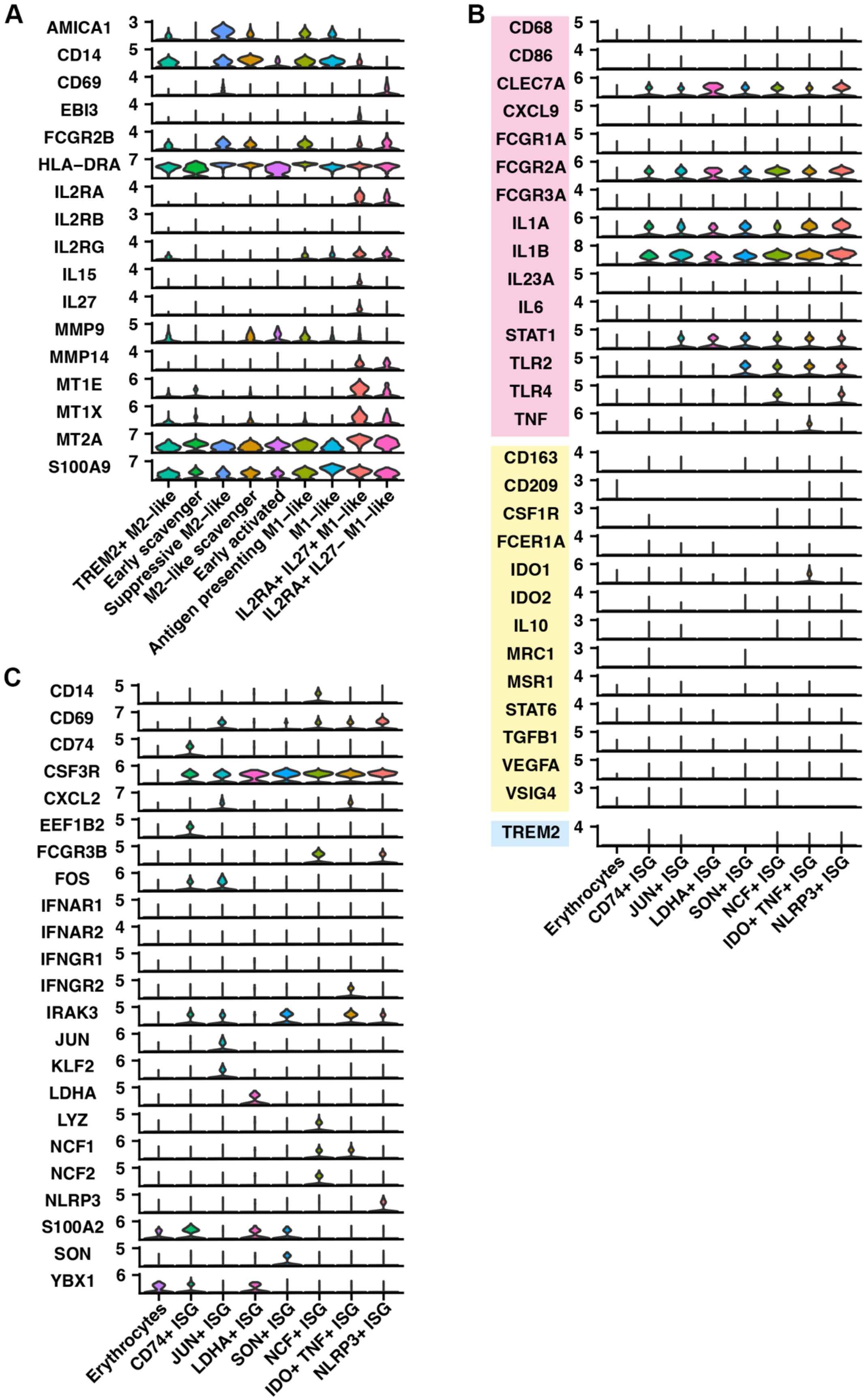

FIG S4 Additional genes differentiating macrophages and ISG-expressing cell subclusters. (**A**) Additional genes expressed by macrophages. (**B**) Macrophage markers expressed by ISG-expressing cells. M1 (pink box), M2 (yellow box), and TREM2 (blue box) markers are shown. (**C**) Additional genes expressed by ISG-expressing cells.

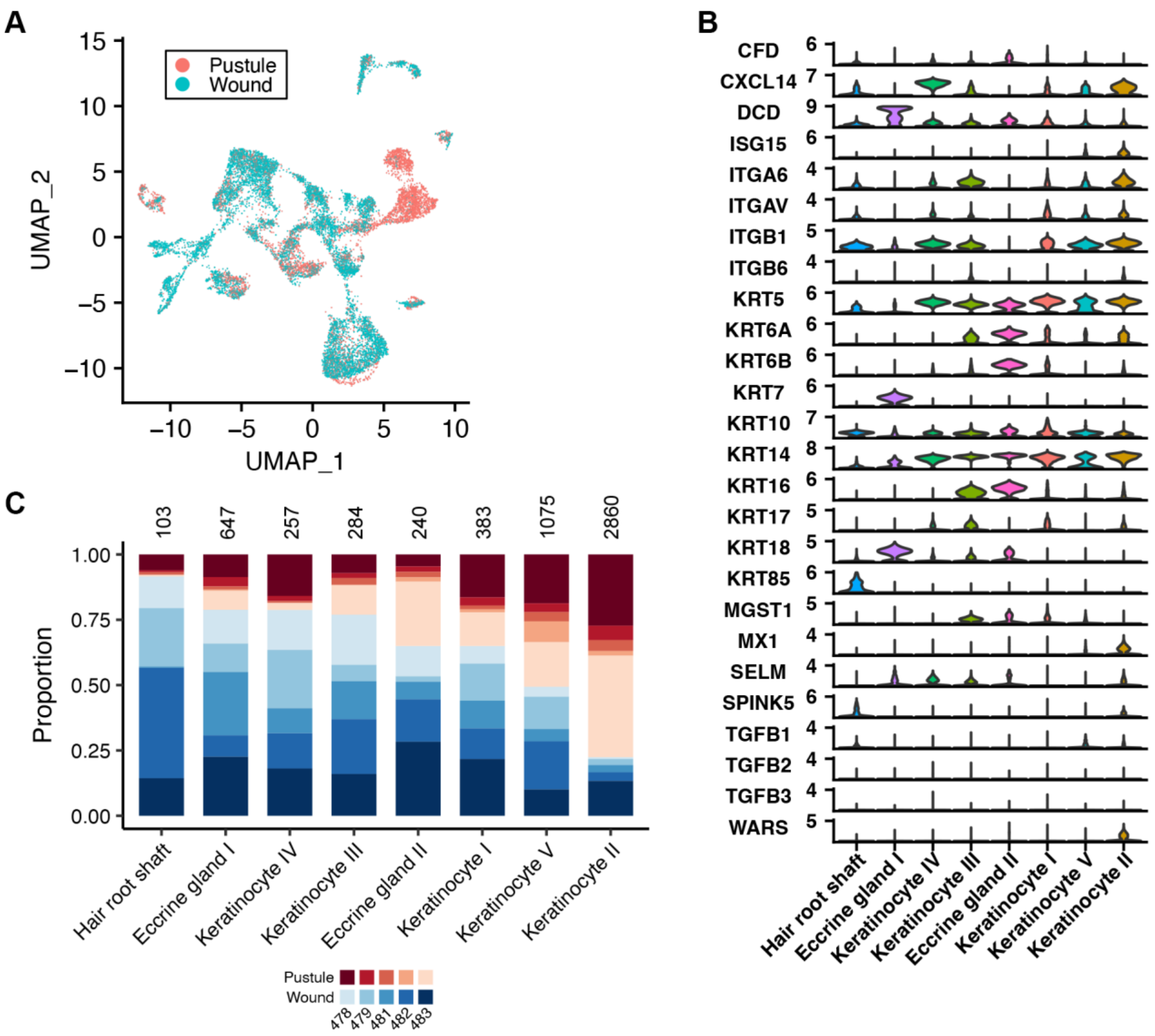

FIG S5 Keratinocytes present in pustules and wounds. (**A**) Dimensional reduction plot showing whether an individual keratinocyte was derived from a pustule or wound sample. (**B**) Violin plot showing the expression of select keratins and immune response-related genes. (**C**) For each cell subset in FIG 6A, the proportion of cells derived from each volunteer is shown. Blue colors indicate cells that were derived from a wound; red colors indicate cells that were derived from a pustule. The different shades of colors indicate the volunteer number. The total number of cells belonging to a subcluster is shown above each bar.

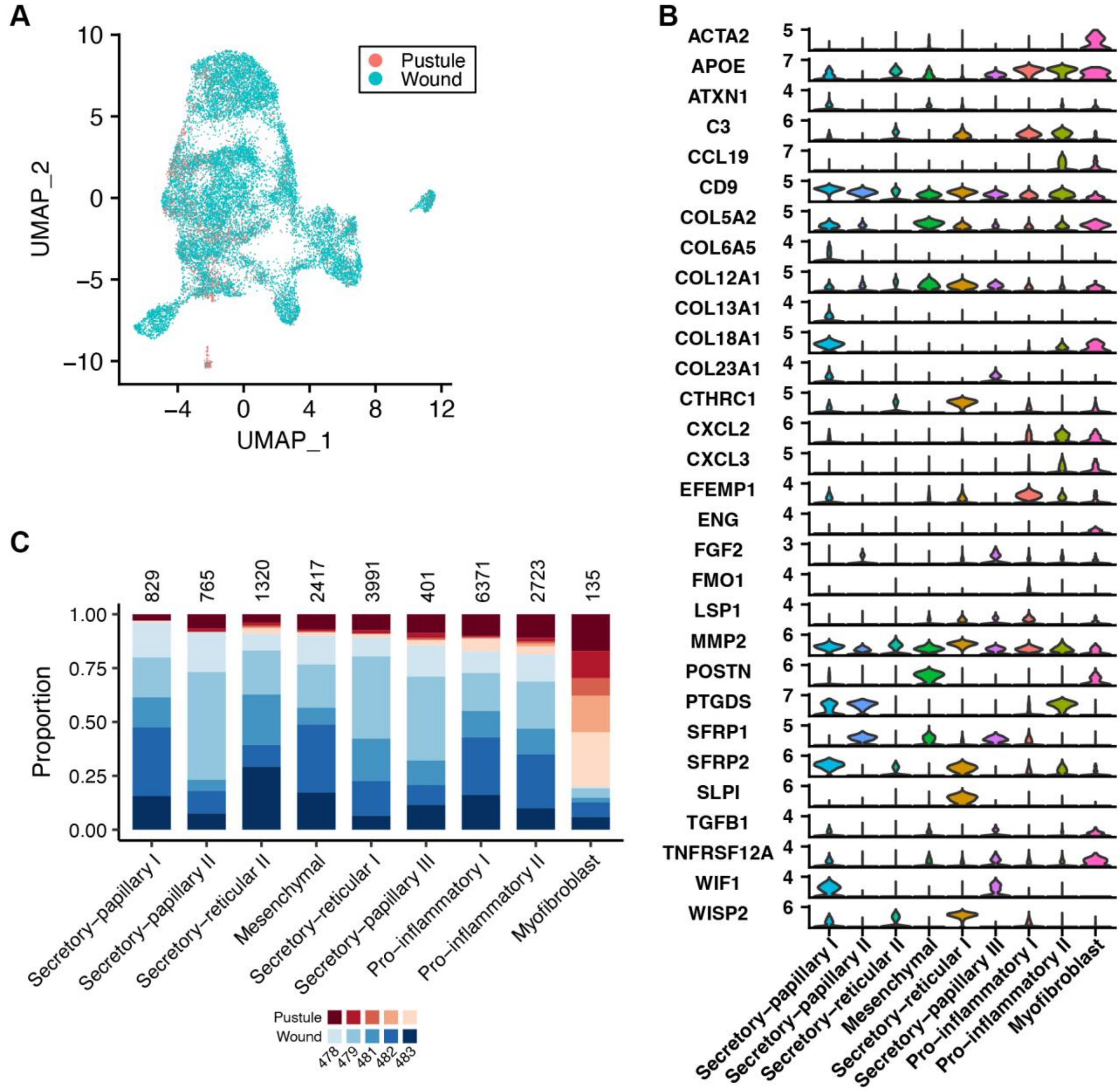

FIG S6 Fibroblasts present in pustules and wounds. (**A**) Dimensional reduction plot showing whether an individual fibroblast was derived from a pustule or wound. (**B**) Violin plot showing the expression of select collagens, fibroblast marker genes, and immune response-related genes. (**C**) For each cell subset in FIG 6C, the proportion of cells derived from each volunteer is shown. Blue colors indicate cells that were derived from a wound; red colors indicate cells that were derived from a pustule. The different shades of colors indicate the volunteer number. The total number of cells belonging to a subcluster is shown above each bar.

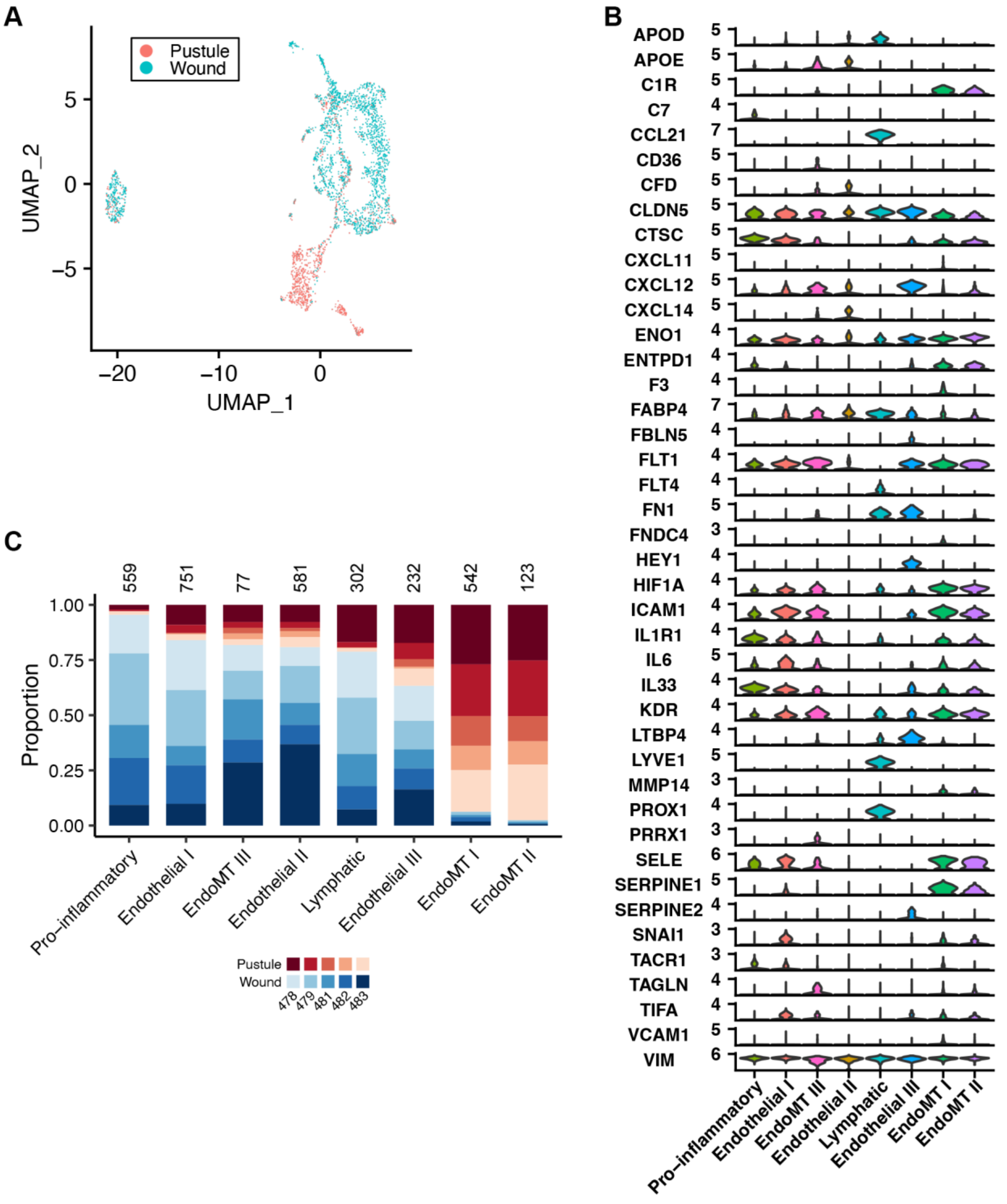

FIG S7 Endothelial cells present in pustules and wounds. (**A**) Dimensional reduction plot showing whether an individual endothelial cell was derived from a pustule or wound. (**B**) Violin plot showing the expression of endothelial cell marker genes and immune response-related genes. (**C**) For each cell subset in FIG 6E, the proportion of cells derived from each volunteer is shown. Blue colors indicate cells that were derived from a wound; red colors indicate cells that were derived from a pustule. The different shades of colors indicate the volunteer number. The total number of cells belonging to a subcluster is shown above each bar.

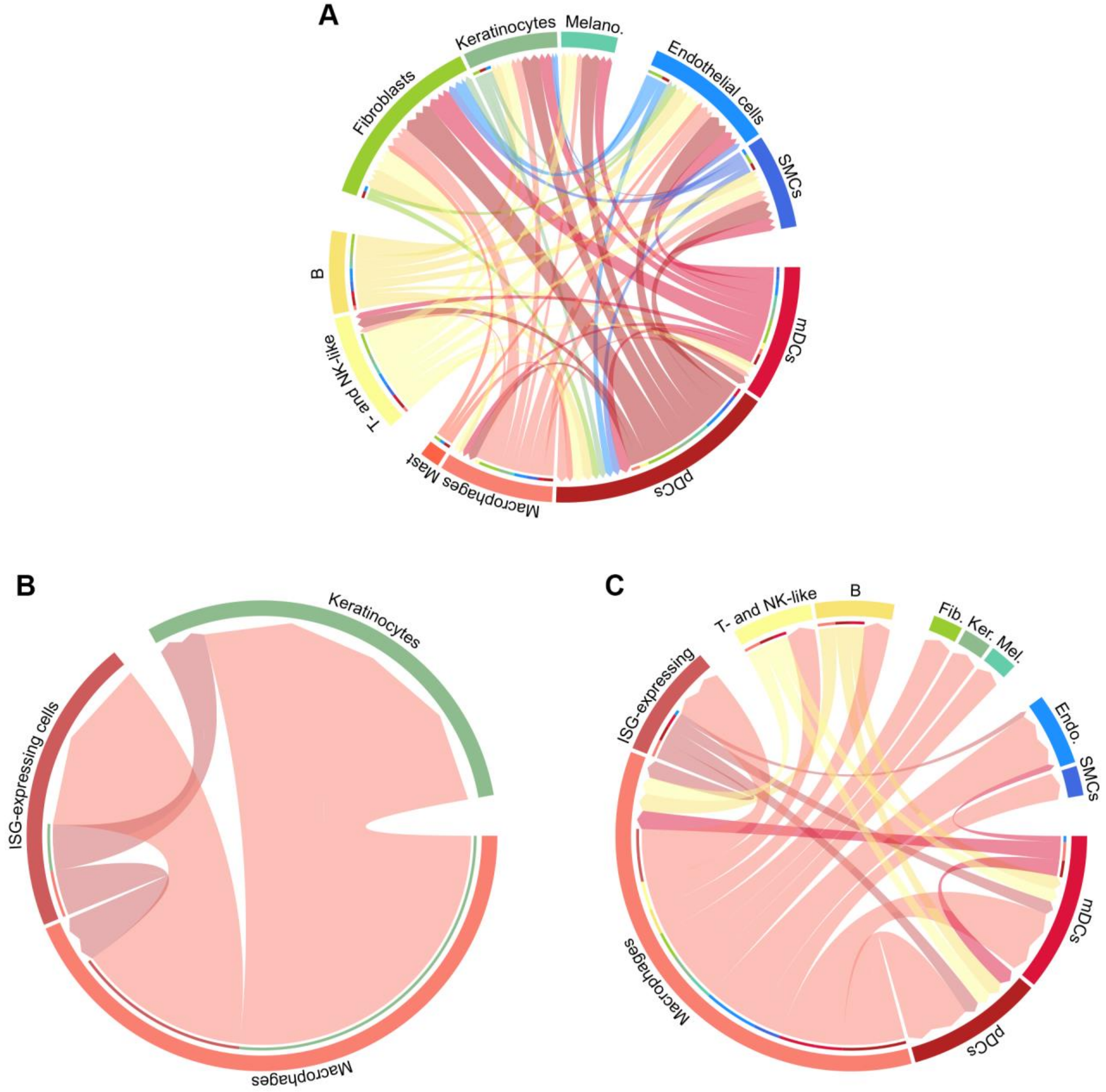

FIG S8 Receptor-ligand analysis of TGF-β, IL-1β, and TNF-α signaling. The interaction strength of select receptor-ligand pairs was analyzed in CellPhoneDB. Circos plots of (**A**) TGF-β, (**B**) IL-1β, and (**C**) TNF-α signaling are shown. The outer ring represents the cellular source of the cytokine; the inner ring represents the cellular source of the receptor(s). Arrow width is proportional to the interaction strength. All major cell clusters were analyzed, but only significant interactions are shown.

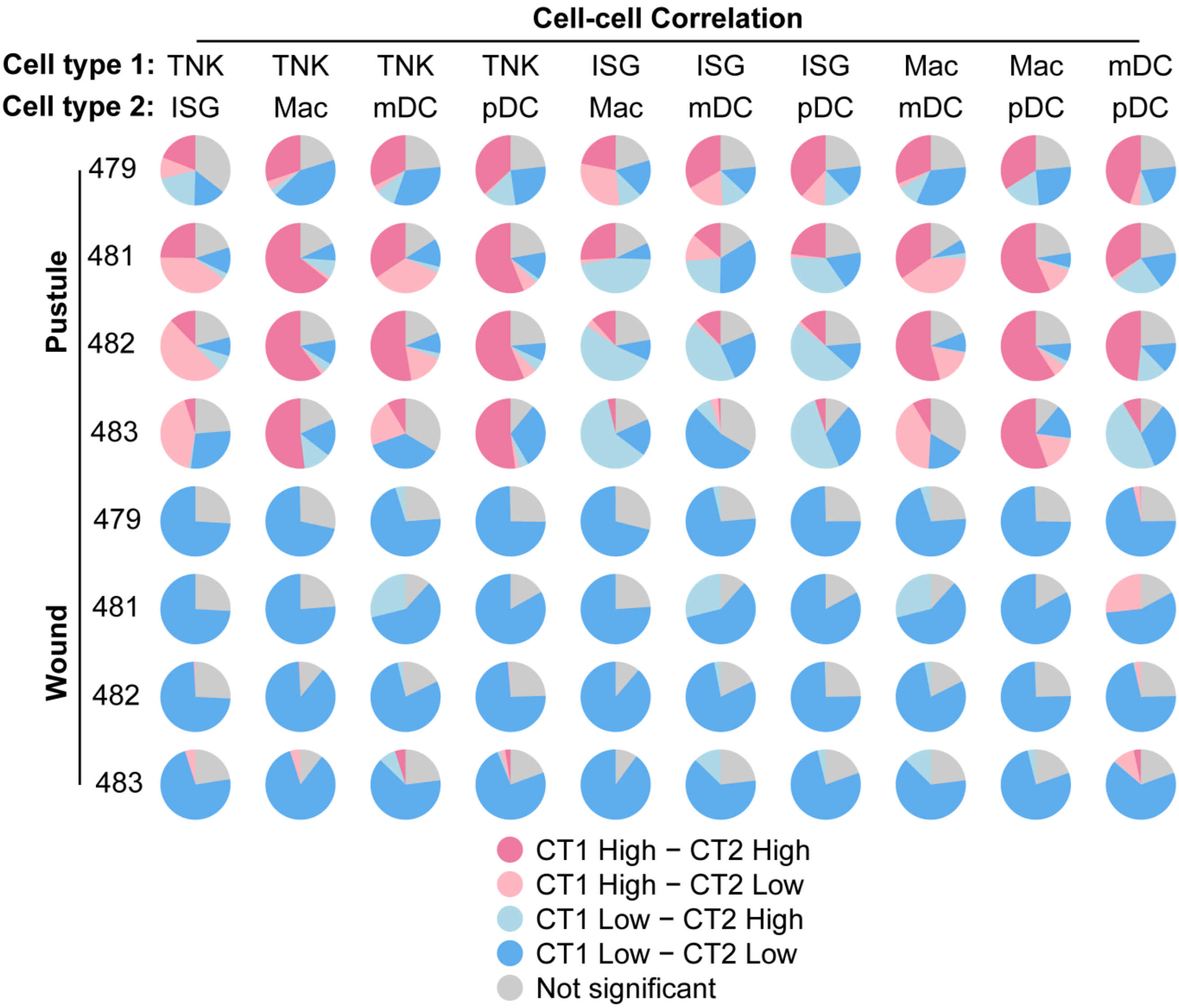

FIG S9 Spatial correlation between different cell types. The spatial pattern of a given major cell type (CT) was compared to the spatial pattern of all other major cell types. The pairs shown are related to antigen presentation. Statistics for all possible cell pairings are in Supplemental Table 1.

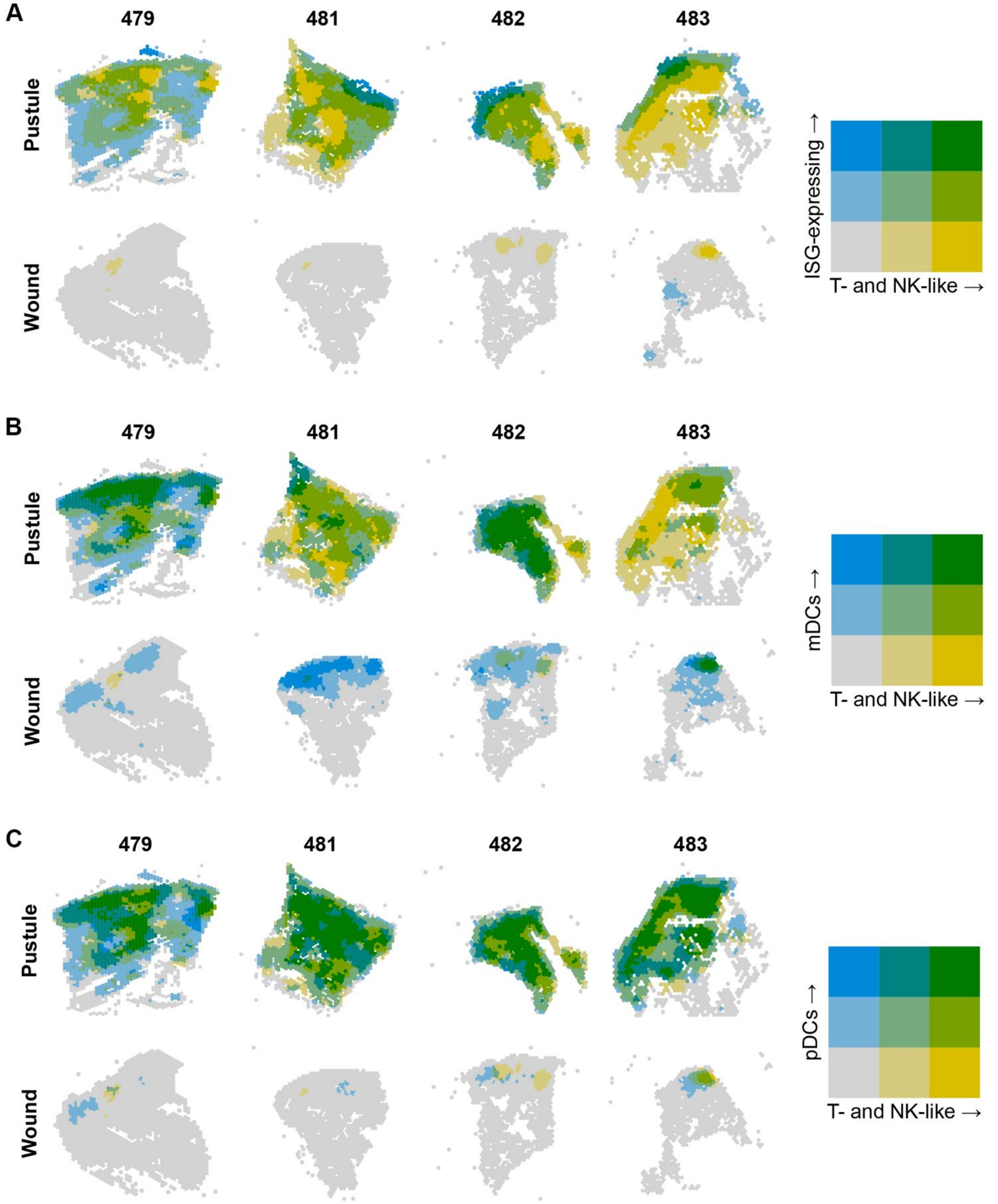

FIG S10 Antigen presentation within pustule and wound sites. Bivariate choropleth maps of the co-occurrence of T- and NK-like cells (yellow) and (**A**) ISG-expressing cells, (**B**) mDCs, or (**C**) pDCs (blue) in each Visium barcode spot. Gray areas lack any signal from either cell type, dark green indicates high signal from both cell types, while blues or yellows indicate that only one cell type is present. Color breaks were determined by normalizing across the pustule-wound pair of each volunteer.

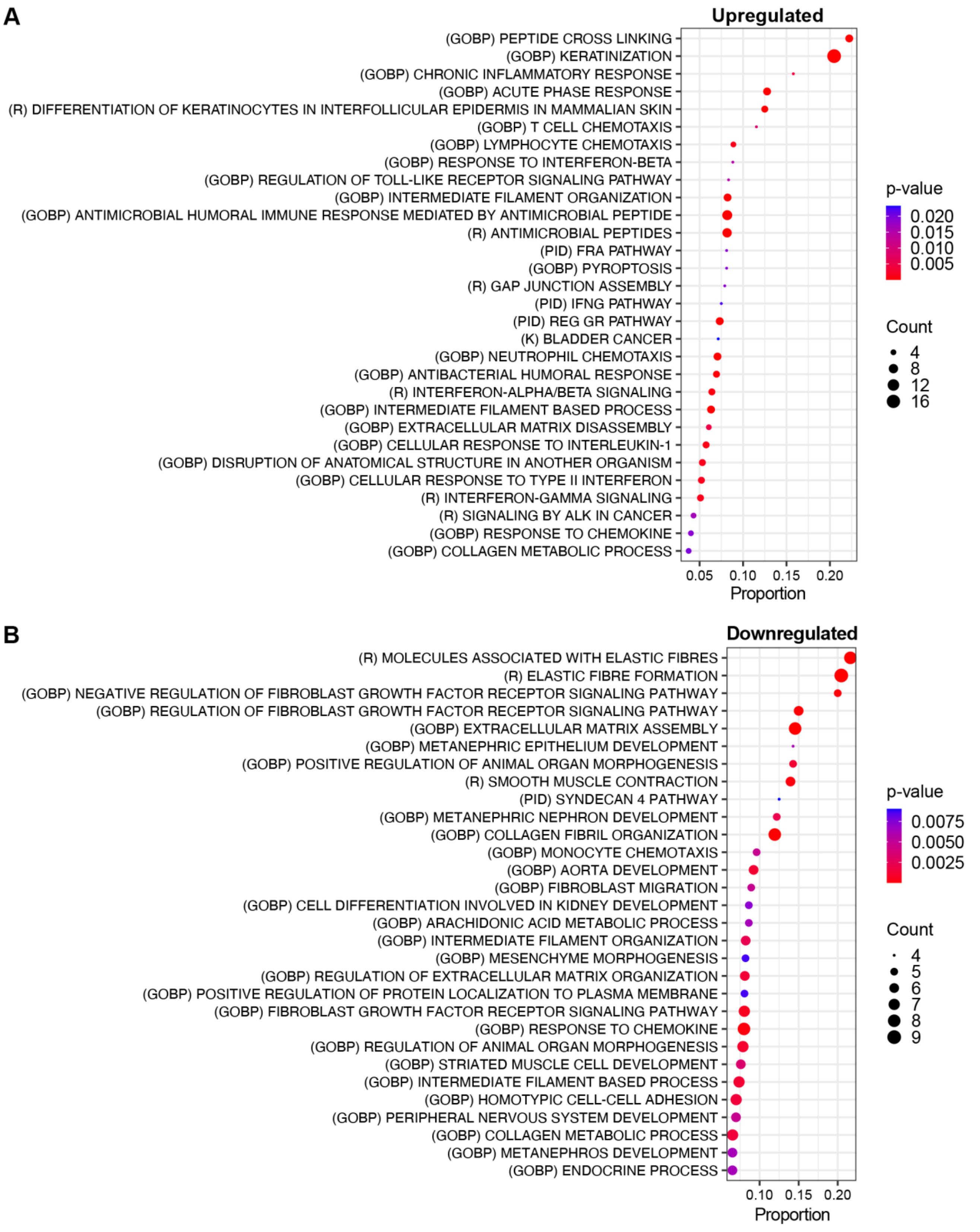

FIG S11 Pathway analysis of differentially expressed genes (DEG) in areas with high TNK-APC association. The top 30 enriched pathways among upregulated (**A**) and downregulated (**B**) genes in pustules. The proportion of genes in the DEG list over the total number of genes in the pathway are shown. The database that each pathway is from is in parentheses. R, Reactome; GOBP, gene ontology biological process; PID, pathway interaction database.
