# Supplementary material for: A high-resolution view of the immune and stromal cell response to Haemophilus ducreyi infection in human volunteers
Source: mBio. 2025 Jan 30;16(3):e03885-24. doi: 10.1128/mbio.03885-24 (PMC11898715; doi:10.1128/mbio.03885-24)
Supplement: Table S1 — Response to inoculation with H. ducreyi 35000HP. [file mbio.03885-24-s0004.docx]

**Table S1.** Response to inoculation with *H. ducreyi* 35000HP

| **Vol. #** | **Race** | **Gender** | **Age (years)** | **EDD(CFU)** | **Days infected** | **# Initial Papules** | **# Pustules at Endpoint** |
| --- | --- | --- | --- | --- | --- | --- | --- |
| 478 | W | M | 27 | 72 | 8 | 3 | 3 |
| 479 | W | F | 27 | 41 | 7 | 3 | 3 |
| 480 | W | F | 26 | 77 | 7 | 3 | 0 |
| 481 | W | M | 46 | 77 | 7 | 3 | 3 |
| 482 | A | M | 28 | 85 | 7 | 3 | 3 |
| 483 | A | M | 38 | 85 | 8 | 3 | 3 |

Notes: Volunteer 478 was infected in the first iteration; 479 in the second iteration; 480 and 481 in the third iteration; 482 and 483 in the fourth iteration. Vol., volunteer; W, white; A, Asian; M, male; F, female; EDD, estimated delivered dose; CFU, colony forming units.
