# Supplementary material for: A high-resolution view of the immune and stromal cell response to Haemophilus ducreyi infection in human volunteers
Source: mBio. 2025 Jan 30;16(3):e03885-24. doi: 10.1128/mbio.03885-24 (PMC11898715; doi:10.1128/mbio.03885-24)
Supplement: Table S3 — Number of single cells before and after filtering and number of spatial spots and average number of genes per spot. [file mbio.03885-24-s0006.docx]

**Table S3.** Number of single cells before and after filtering and number of spatial spots and average number of genes per spot from the spatial transcriptomics data.

|  | **Single cells before filtering** | **Single cells after filtering** | **Number of spatial spots** | **Average number of genes per spot** |
| --- | --- | --- | --- | --- |
| 478 Pustule | 12735 | 8331 | ND | ND |
| 478 Wound | 9344 | 6183 | ND | ND |
| 479 Pustule | 6735 | 4161 | 1895 | 1670 |
| 479 Wound | 16853 | 9921 | 1326 | 978 |
| 481 Pustule | 5990 | 4733 | 1475 | 5711 |
| 481 Wound | 9606 | 6418 | 1064 | 3012 |
| 482 Pustule | 12650 | 10045 | 917 | 9691 |
| 482 Wound | 14840 | 11329 | 950 | 5585 |
| 483 Pustule | 18826 | 12633 | 1117 | 6281 |
| 483 Wound | 12726 | 9218 | 773 | 3455 |

Note: ND, not determined.
